# The impact of labeling automotive AI as trustworthy or reliable on user evaluation and technology acceptance

**DOI:** 10.1038/s41598-025-85558-2

**Published:** 2025-01-09

**Authors:** John Dorsch, Ophelia Deroy

**Affiliations:** 1 Faculty of Philosophy, Philosophy of Science and the Study of Religion, Ludwig-Maximilians-Universität München, Munich, Germany; 2 Munich Center for Neurosciences, Ludwig-Maximilians-Universität München, Munich, Germany; 3 Institute of Philosophy, School of Advanced Study, University of London, London, UK; 4 Institute of Philosophy, Czech Academy of the Sciences, Prague, Czech Republic

**Keywords:** Trustworthy AI, Ethics of AI, Human–machine interface, Technology acceptance, Algorithm aversion, Human behaviour, Information technology

## Abstract

This study explores whether labeling AI as either “trustworthy” or “reliable” influences user perceptions and acceptance of automotive AI technologies. Utilizing a one-way between-subjects design, the research presented online participants (N = 478) with a text presenting guidelines for either trustworthy or reliable AI, before asking them to evaluate 3 vignette scenarios and fill in a modified version of the Technology Acceptance Model which covers different variables, such as perceived ease of use, human-like trust, and overall attitude. While labeling AI as “trustworthy” did not significantly influence people’s judgements on specific scenarios, it increased perceived ease of use and human-like trust, namely benevolence, suggesting a facilitating influence on usability and an anthropomorphic effect on user perceptions. The study provides insights into how specific labels affect adopting certain perceptions of AI technology.

## Introduction

The widespread use of the “trustworthy AI” denomination by manufacturers and legislators sparks debates. On the one hand, several philosophers have argued that it is irrational to place trust in a machine^1–4^. Instead, trust should be placed not in the machines themselves, but in the humans responsible for their development and deployment^5^. Others, like Coeckelbergh^6^ for instance, argue in favor of trusting machines, emphasizing that as social animals, we already live in a world structured by trusting relationships. Accordingly, trust should be considered a pre-reflective condition of human experience and unproblematically extended to machines.

Those who argue against trusting machines contend that trust can only be rationally employed if the trustee is sensitive to normative constraints of reasons^3^, has the trustee’s goodwill in mind when acting^7^, holds the trustee’s interests as their own^8^, or is emotionally sensitive to moral values^9^. Since today’s machines are nowhere near any of these conditions, many AI ethicists advocate for employing the notion of reliability rather than trust when dealing with machines. Reliability, decoupled from trust, is understood as entailing solely performance-based or outcome-based standards for evaluation^2,3^.

However, despite the theoretical divergence between trust and reliability, their practical effect on user expectations and/or criteria of evaluation might diverge when engaging with AI technologies. Reliability here refers to a focus on whether the AI system consistently performs as expected, without necessarily attributing human-like qualities to the machine. In contrast, trust involves a deeper expectation of moral sensitivity and goodwill, which AI systems are not equipped to meet. This difference in frameworks for conceiving one’s relationship to machines-the framework of trust and the framework of reliability-raises an important question on attributions of responsibility.

In specific scenarios where AI technologies lead to a poor outcome, humans tend to hold machines responsible, that is, blame and praise them^10–12^. Given that these responses are anthropocentric and have social implications, it raises the question of whether individuals will be less inclined to hold AI accountable when applying a reliability-based framework as opposed to a trust-based one. This study aimed to investigate whether the adoption of either framework-trust-based, which emphasizes moral responsibility and goodwill, or reliability-based, which focuses on performance and outcome-based evaluation-would influence the perceptions of naive users regarding special scenarios linked to automotive AI. Specifically, we tested whether these differing frameworks would lead to variations in users’ evaluations of AI in automotive contexts.

When it comes to technology acceptance, empirical investigation often relies on the Technology Acceptance Model (TAM). The TAM framework helps in understanding how users come to accept and use a technology, focusing on distinct factors such as perceived ease of use, perceived usefulness, and attitude towards the technology. In our study, we employed a streamlined TAM based on the constructs identified by Davis^13^ and further refined by Venkatesh and Davis^14^, as well as insights from Choung et al.^15^, to assess whether either the trust or reliability framework would predict changes in these variables. This approach allowed us to measure whether these frameworks would influence overall technology acceptance or specific aspects.

Previous studies investigating user perception of automotive AI have had as their focus anthropomorphized autonomous vehicles^16–19^, showing that enhanced anthropomorphization can increase perceived trust in this technology (cf.^20^). Anthropomorphizing likely skews participant responses toward trustworthiness, since the more anthropomorphized the autonomous car is, the more inclined the participant will be to treat the technology like a human-like agent. For this reason, this study seeks to understand perceptions toward AI assistance in driving, wherein it is arguably more appropriate to conceive of AI as a tool.

Finally, understanding the effects of labels on technology acceptance is worthwhile, as these can offer insight into how to mitigate algorithm aversion or exploitation-phenomena where users reject AI technology despite its potential to enhance performance or collective benefits^20–22^.

### Hypotheses

In this study, we investigated the impact of labeling AI as “trustworthy” versus “reliable” on various dependent variables related to technology attitudes. Our hypotheses (H1, H2, H3, H4, H5) examine whether the label “trustworthy AI” predicts changes in AI blameworthiness, AI accountability, confidence while using, and confidence while learning, and the overall TAM score. Additionally, we explored whether the labels predict changes in TAM categories, including perceived ease of use, perceived usefulness, intention to use, ability-based and human-like trust in automotive AI (H5.1–H5.8) (see Table 1). The study was pre-registered to enhance transparency and credibility. The hypotheses remain consistent with those outlined in the pre-registration, though the numbering has been reordered and simplified, the null hypotheses are reformulated as “no predicted effect”. The pre-registration document can be accessed at the Open Science Framework (OSF) via this link.

## Results

The results of the hypothesis testing went against most pre-registered hypotheses, which were based on the literature as well as the pilot with 43 participants, 21 in the “trustworthy AI” group and 22 in the ”reliable AI” group. For hypotheses H1, H2, which predicted that the trustworthy AI condition would lead to lower ratings of blameworthiness and accountability, we did not observe any effect and could therefore not reject the null hypothesis. For Hypotheses H3, and H4, which concerned confidence ratings and where no special prediction was issued, no effect was observed. Finally, for Hypothesis H5, predicting a lower total TAM score, we could not reject the null hypothesis as we did not observe any significant difference.

Moving to specific aspects of trustworthy AI’s impact, the results indicate a significant effect of the label “trustworthy AI” on two of the TAM variables: higher ratings of H5.1 perceived ease of use and H5.5 benevolence, both of which differ from preregistration, but suggest that the conditions did affect some aspects of technological acceptance. The other hypotheses did not reach significance. For a comprehensive overview of these results, please refer to Table 1.

**Table 1:**
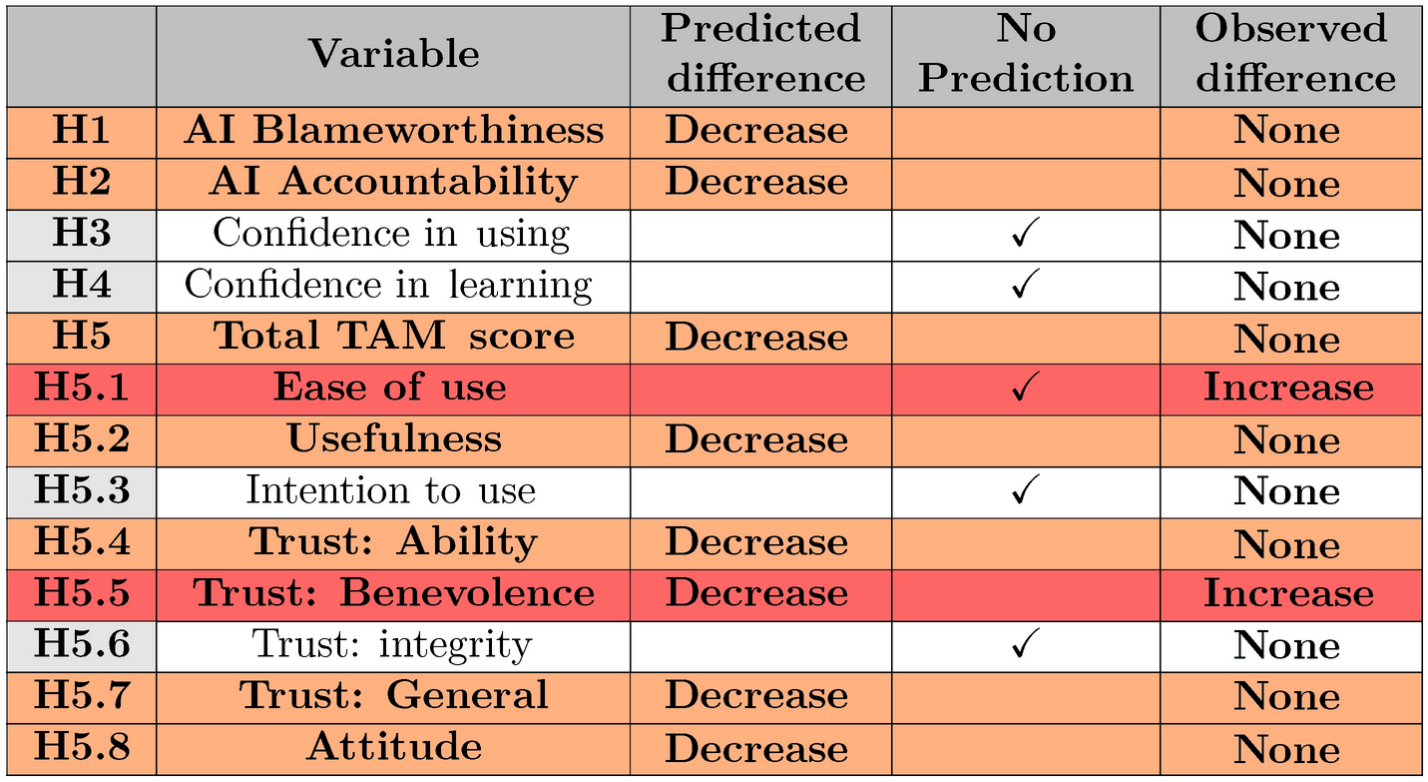
Hypotheses and results overview.

Each hypothesis pertains to the label “trustworthy AI” and its potential effect on the variable (increase or decrease). Red lines indicate an observed effect that was different from the predicted effect. Orange indicates that the null hypothesis could not be rejected. No predicted effects were observed

### Vignette responses: H1–H4

**Fig. 1:**
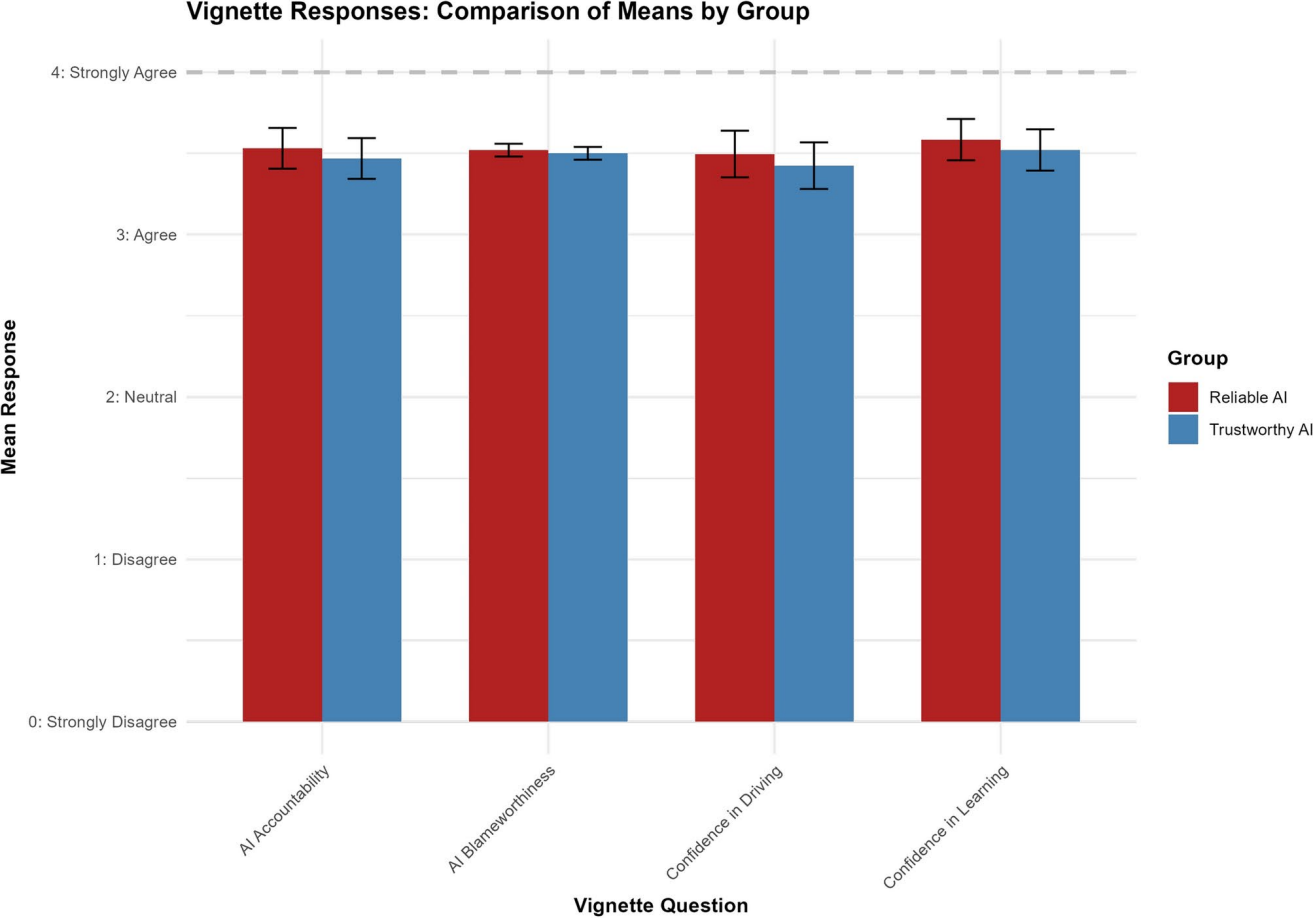
Results for vignette responses: H1–H4.

As detailed in the pre-registered plan, we conducted t-tests to compare group means on AI accountability, AI blameworthiness, confidence in driving, and confidence in learning between the trustworthy AI and reliable AI groups (See Fig. 1). The analysis for each hypothesis showed that there were no significant differences between the conditions: $p$ values for all comparisons were greater than $0.05$, indicating that the observed differences were not statistically significant, as confirmed by Bayesian ordinal regression (see Supplementary Materials).

#### H1: AI accountability

There was no significant difference between the trustworthy AI group (mean = $3.47$) and the reliable AI group (mean = $3.53$) in terms of accountability, $t(478) = 1.05$, $p = 0.292$, with a 95% confidence interval of $[-0.0541, 0.180]$.

#### H2: AI blameworthiness

The blameworthiness ratings were similar for the trustworthy AI group (mean = $3.50$) and the reliable AI group (mean = $3.52$), $t(478) = 0.333$, $p = 0.739$, with a 95% confidence interval of $[-0.0963, 0.136]$.

#### H3: Confidence in driving

Participants’ confidence in driving using the automotive AI did not significantly differ between the trustworthy AI group (mean = $3.42$) and the reliable AI group (mean = $3.50$), $t(478) = 1.264$, $p = 0.207$, with a 95% confidence interval of $[-0.0398, 0.184]$.

#### H4: Confidence in learning

Similarly, confidence in learning to drive with the automotive AI was not significantly different between the trustworthy AI group (mean = $3.52$) and the reliable AI group (mean = $3.59$), $t(478) = 1.07$, $p = 0.285$, with a 95% confidence interval of $[-0.0531, 0.180]$.

#### Jitter plot analysis of vignette data

**Fig. 2:**
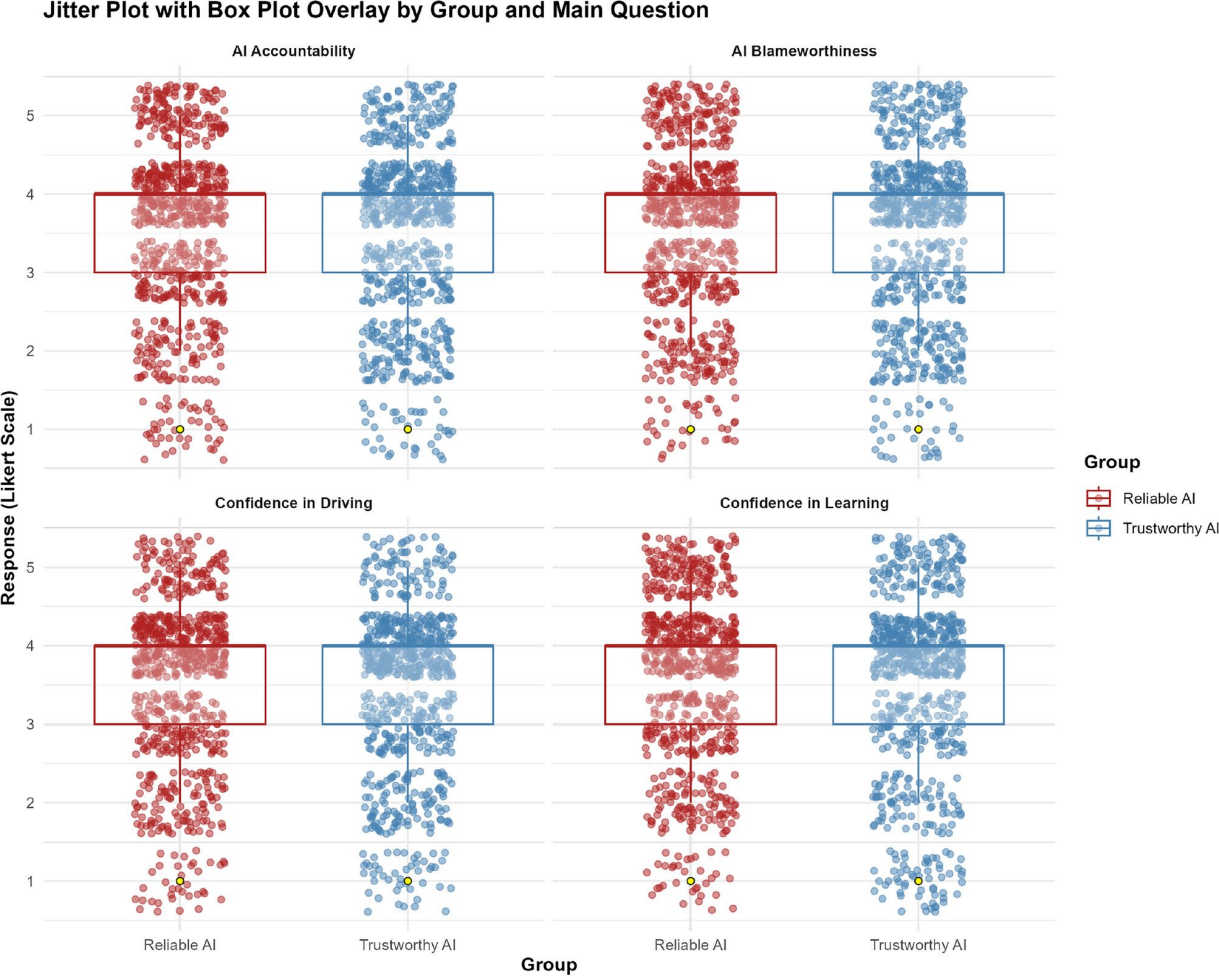
Jitter plot with box plot overlay for the vignette responses.

The jitter plot with box plot overlay illustrates the distribution of responses across four key measures (See Fig. 2). The box plots, which summarize the central tendency and spread of the responses, show that the medians and interquartile ranges (IQRs) are similar across both conditions. The overlapping IQRs in the box plots indicate that participants in both conditions exhibit similar attitudes and perceptions.

### Total technology acceptance score: H5

**Fig. 3:**
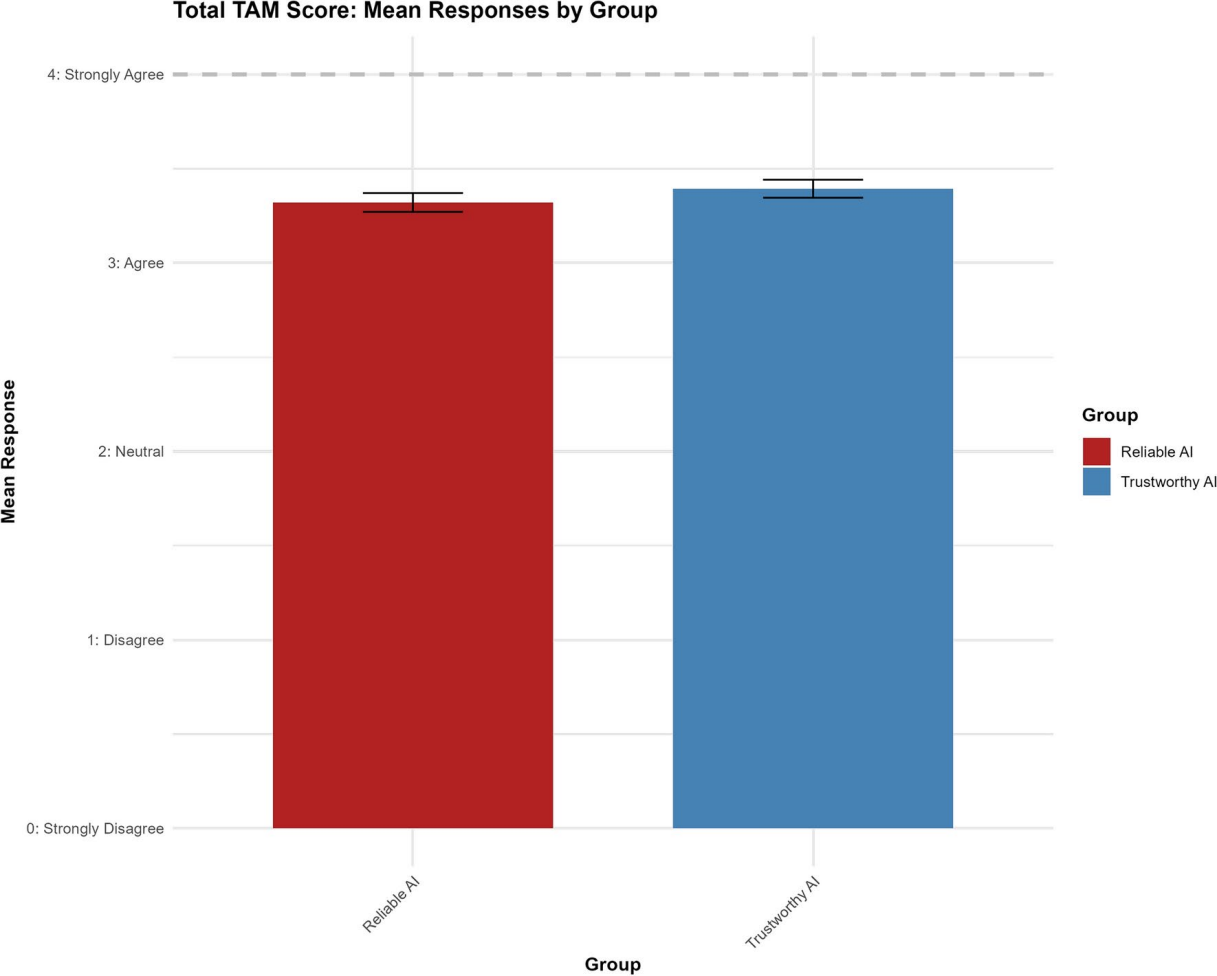
Results for total TAM score.

To examine the relationship between the condition and participants’ overall perceptions on automotive AI, we conducted ordinal regression analyses on the Total TAM Score (see Fig. 3). The model showed no statistically significant effect of the “trustworthy AI” label, with a coefficient for the grouptrust variable of $0.2058$ $(p = 0.335)$ and confidence intervals ranging from $-0.2131$ to $0.6247$. Thresholds for the response categories were also non-significant. The Brant test confirmed that the parallel regression assumption held $(\chi ^{2}(3) = 7.11, p = 0.07)$. These results suggest no significant impact of the condition on the Total TAM Score.

### Individual technology acceptance items: H5.1–H5.8

**Fig. 4:**
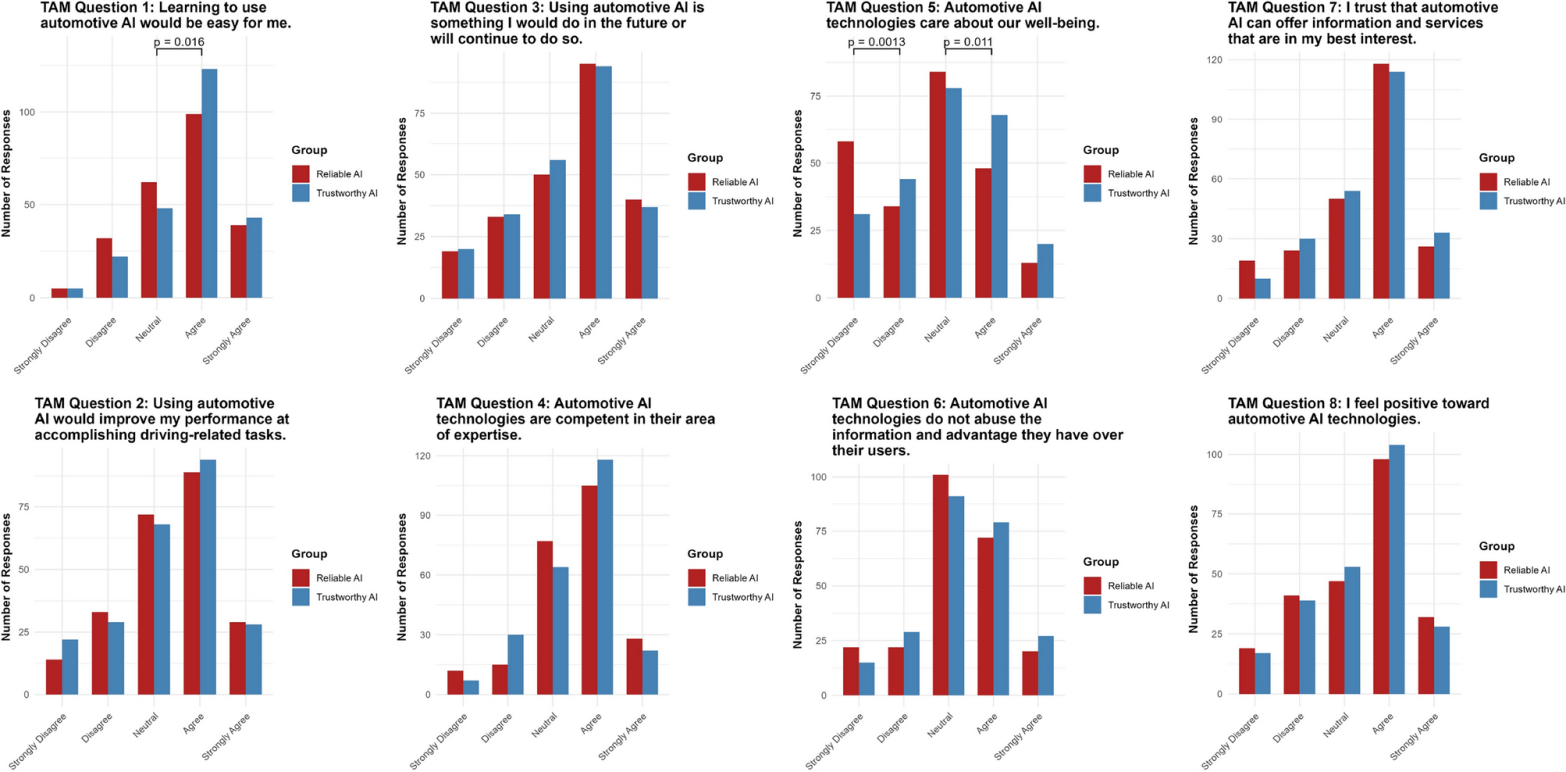
Results for H5.1–H5.8: all eight TAM questions.

For the eight individual TAM items (see Fig. 4), we conducted ordinal regression analyses to examine the influence of the group-specific label. Preliminary tests indicated that not all items met the proportional odds assumption (see Supplementary Materials for details of the Brant test). To ensure a consistent analytic approach, we employed models with flexible thresholds for all items. Below, we present the key results for each question, with full model diagnostics, coefficients, and threshold estimates provided in the Supplementary Materials.

#### Question 1: Learning to use automotive AI would be easy for me

**Fig. 5:**
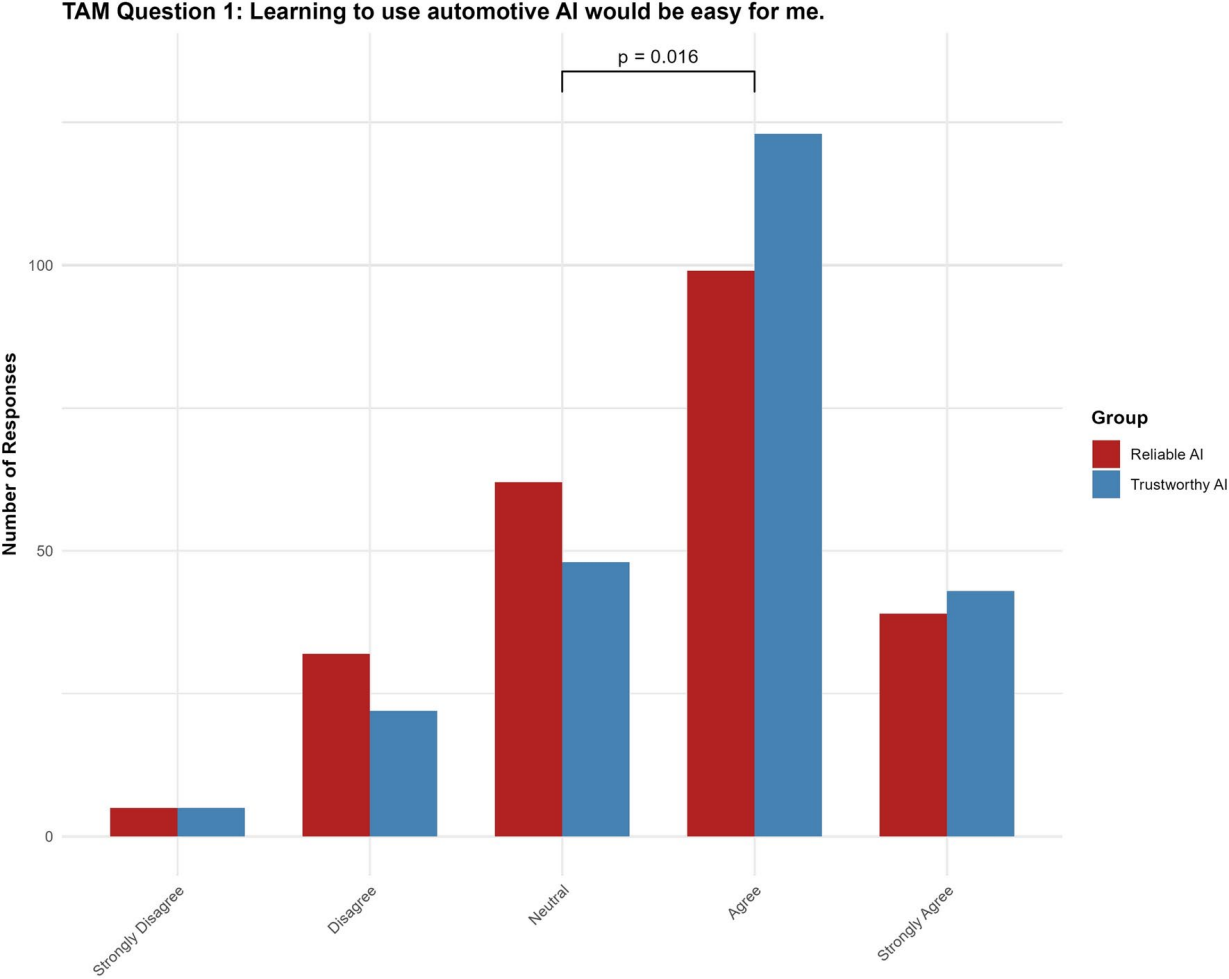
Results for TAM question 1.

For Question 1, the “trustworthy AI” label had a significant positive effect ( $p = 0.0481$, 95% CI $[0.0028, 0.6679]$ ). Additionally, the Neutral|Agree threshold difference reached significance ($p = 0.016$ (see Fig. 5)).

#### Question 2: Using automotive AI would improve my performance at accomplishing driving-related tasks

**Fig. 6:**
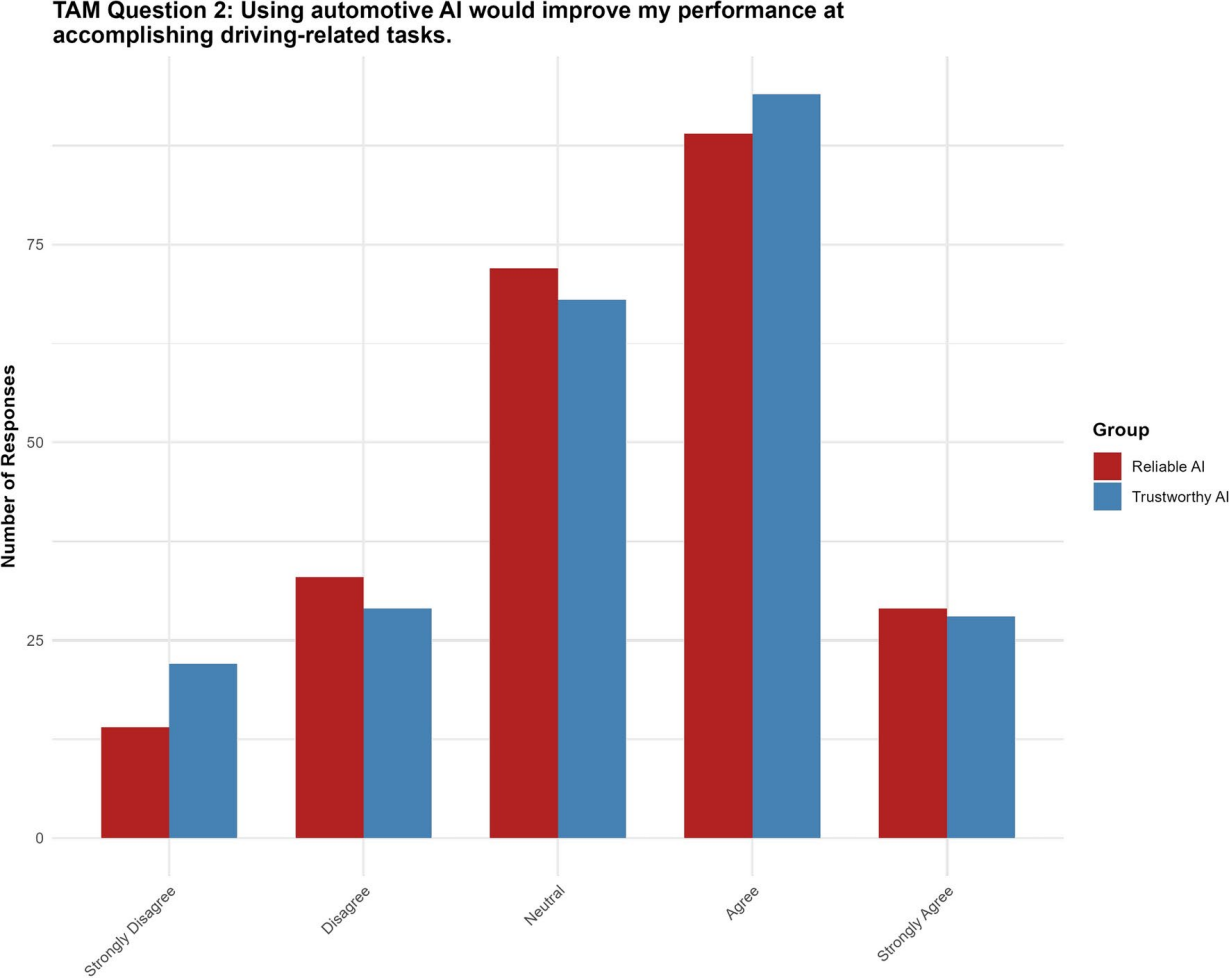
Results for TAM question 2.

For Question 2, the “trustworthy AI” label had no significant effect ($p = 0.848$, 95% CI $[-0.3566, 0.2929]$). None of the thresholds reached statistical significance (see Fig. 6).

#### Question 3: Using automotive AI is something I would do in the future or will continue to do so

**Fig. 7:**
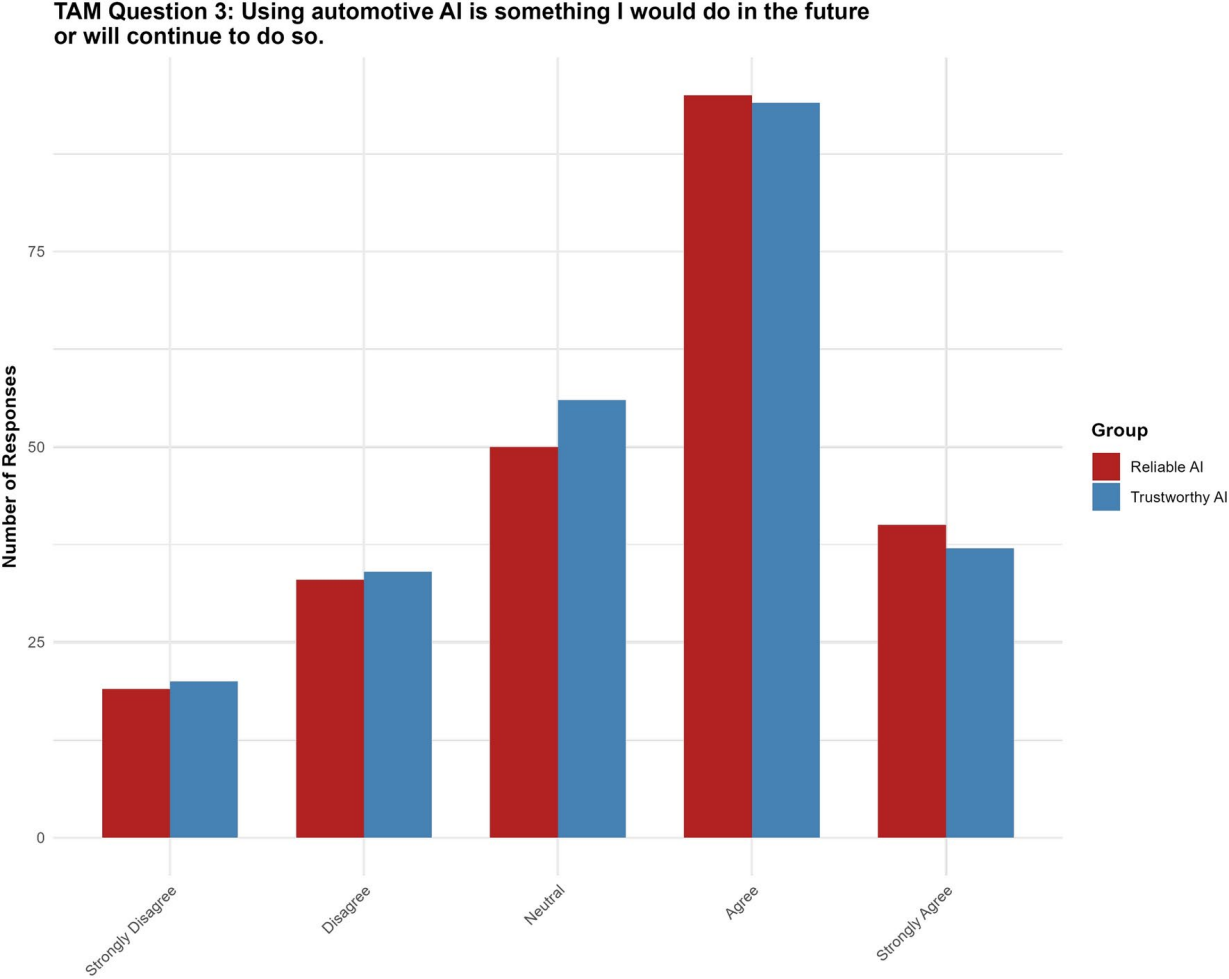
Results for TAM question 3.

For Question 3, the “trustworthy AI” label had no significant effect ($p = 0.596$, 95% CI $[-0.4116, 0.2363]$) on participants’ intention to use the technology. None of the thresholds reached statistical significance (see Fig. 7).

#### Question 4: Automotive AI technologies are competent in their area of expertise

**Fig. 8:**
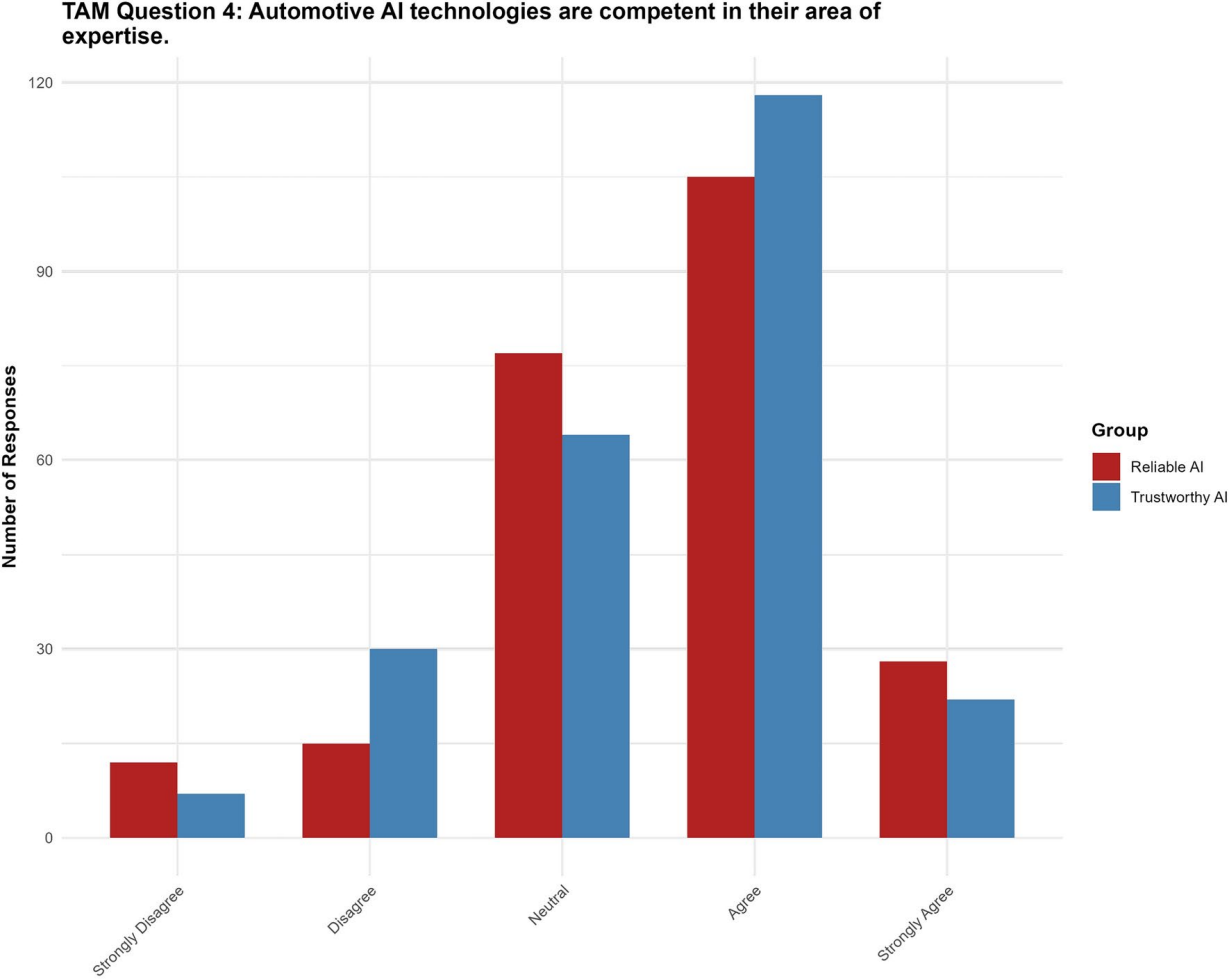
Results for TAM question 4.

For Question 4, the “trustworthy AI” label had no significant effect ($p = 0.795$, 95% CI $[-0.3771, 0.2886]$) on the perception of competence. None of the thresholds reached statistical significance (see Fig. 8).

#### Question 5: Automotive AI technologies care about our well-being

**Fig. 9:**
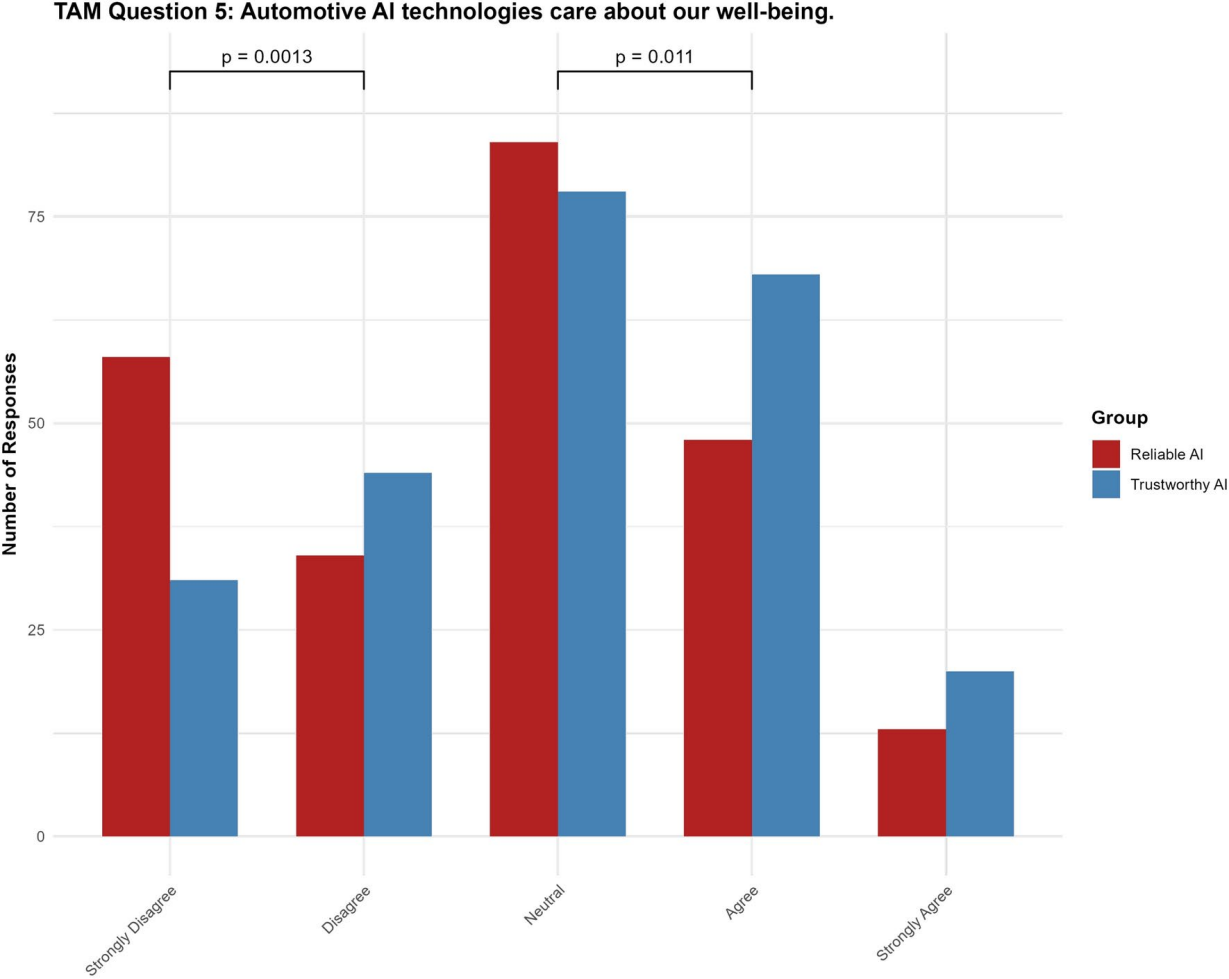
Results for TAM question 5.

For Question 5, the “trustworthy AI” label had a significant positive effect ($p = 0.00323$, 95% CI $[0.1626, 0.8097]$) on the perception that automotive AI cares about users’ well-being. Additionally, the Strongly Disagree|Disagree $(p = 0.0013)$ and Neutral|Agree $(p = 0.011)$ threshold differences were significant (see Fig. 9).

#### Question 6: Automotive AI technologies do not abuse the information and advantage they have over their users

**Fig. 10:**
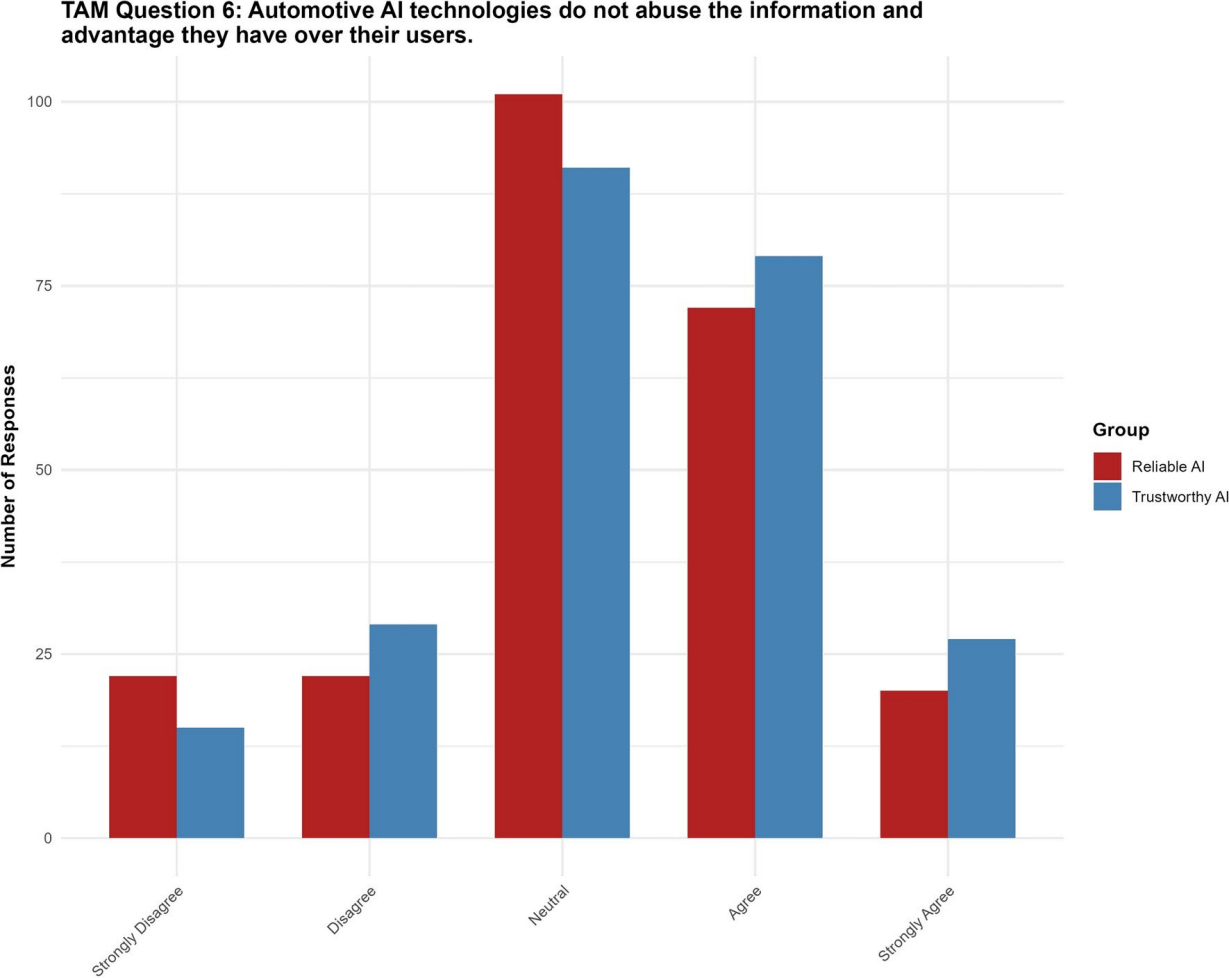
Results for TAM question 6.

For Question 6, the “trustworthy AI” label had no significant effect ($p = 0.267$, 95% CI $[-0.1419, 0.5132]$) on the perception of information abuse. None of the thresholds reached statistical significance (see Fig. 10).

#### Question 7: I trust that automotive AI can offer information and service that is in my best interest

**Fig. 11:**
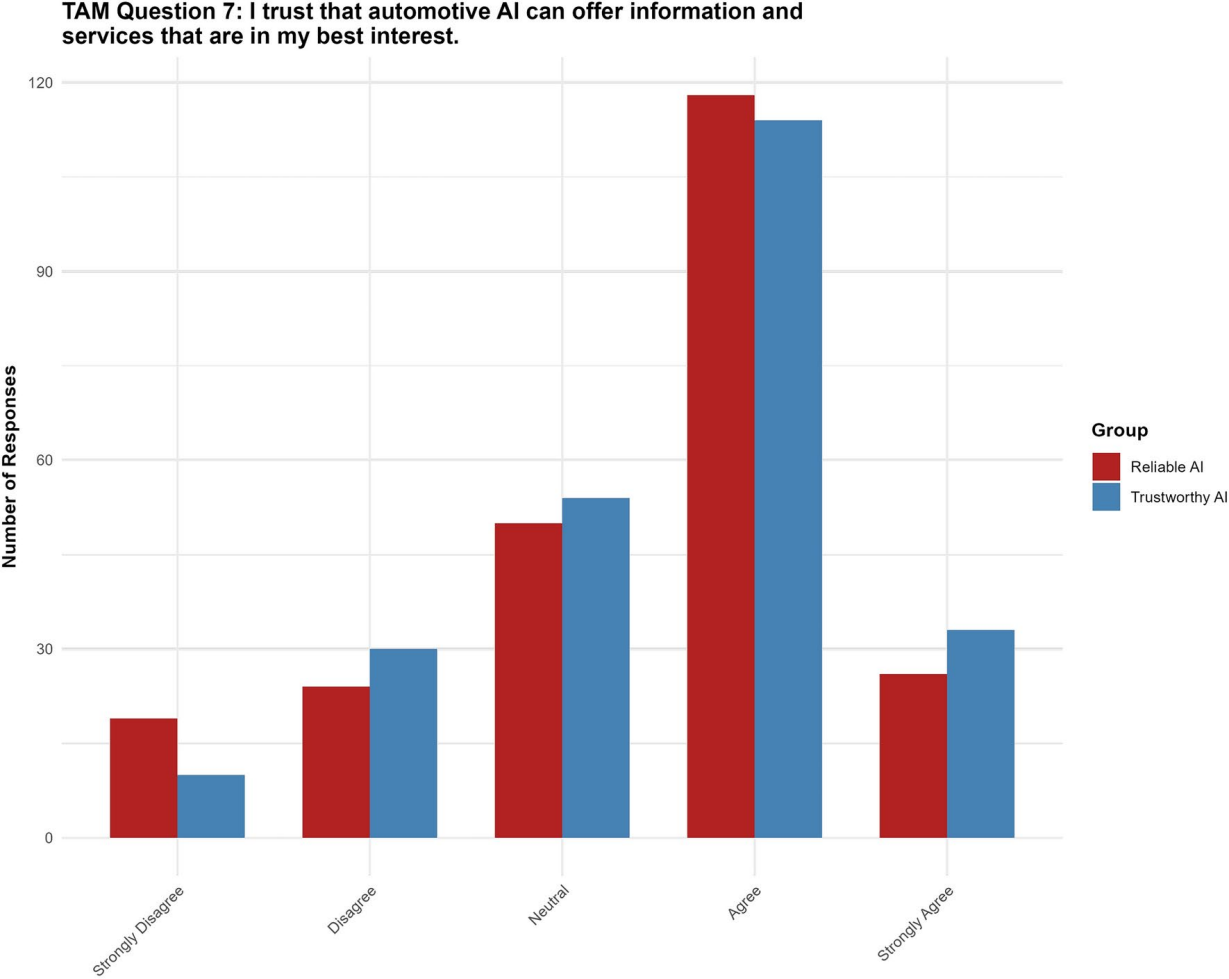
Results for TAM question 7.

For Question 7, the “trustworthy AI” label had no significant effect ($p = 0.542$, 95% CI $[-0.2292, 0.4362]$) on the perception that automotive AI acts in the user’s best interest. None of the thresholds reached statistical significance (see Fig. 11).

#### Question 8: I feel positive toward automotive AI technologies

**Fig. 12:**
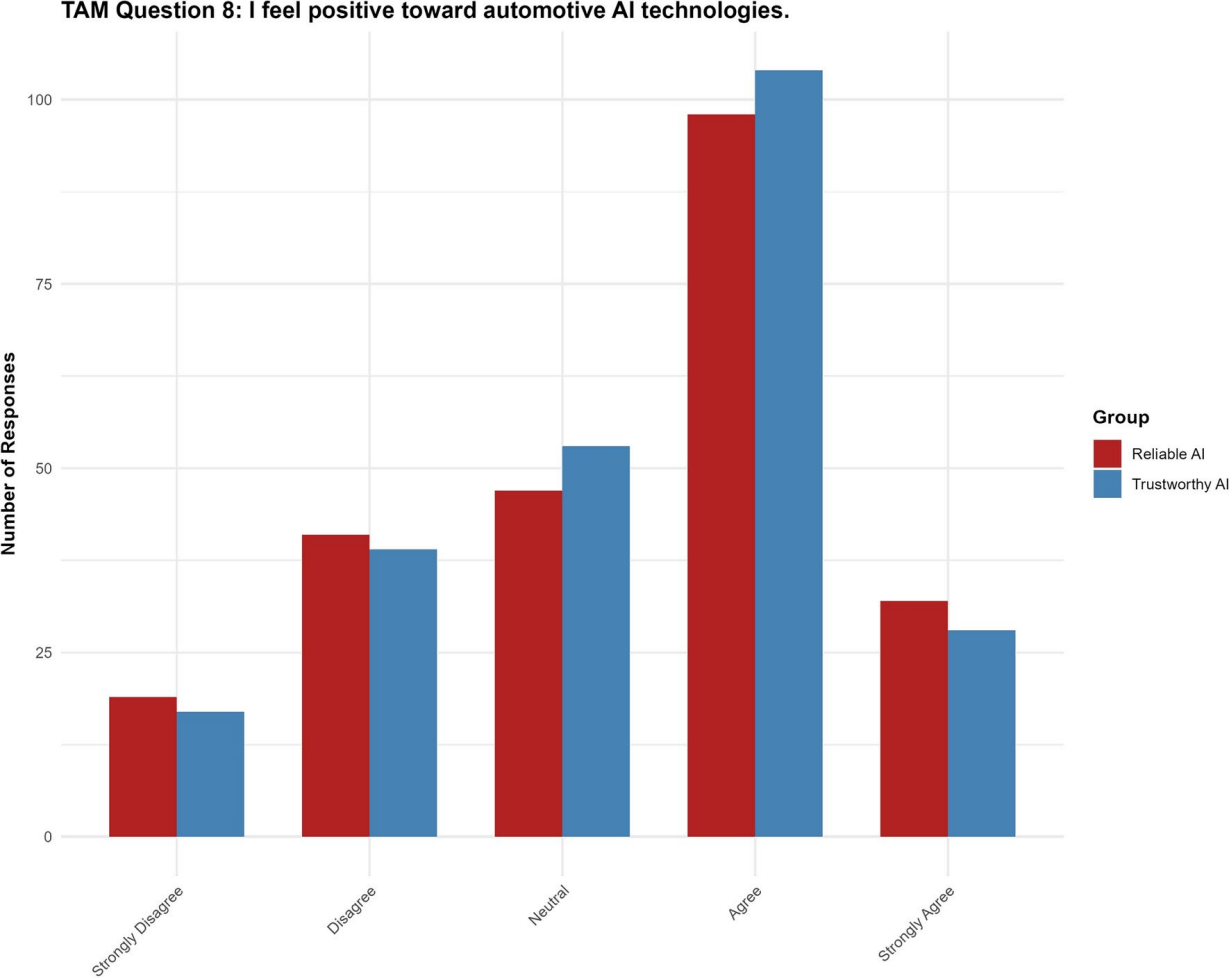
Results for TAM question 8.

For Question 8, the “trustworthy AI” label had no significant effect ($p = 0.989$, 95% CI $[-0.3282, 0.3238]$) on participants’ positive perceptions of automotive AI. None of the thresholds reached statistical significance (see Fig. 12).

## Discussion

This study aimed to investigate the impact of labeling AI as “trustworthy” versus “reliable” on user perceptions and acceptance of automotive AI technologies. Regarding specific vignette scenarios (H1–H4), the study found no significant differences between these conditions. Users were neither more lenient nor more judgmental when AI was labeled as “trustworthy” or “reliable” in terms of accountability and blameworthiness. The same held for confidence in using and learning to use the technology. Although these findings go opposite to the prediction, they suggest that nothing is gained especially in terms of confidence by using an anthropomorphic “trustworthy” label.

The other focus of the study was on the Technology Acceptance Model, exploration of Total TAM score (H5) and sub-categories (H5.1–H5.8). Although no differences were observed in the total TAM score, the results suggest that the “trustworthy” label may improve perceived ease of use and increase the perception that AI cares about the well-being of people. In particular, this label appears to lead to anthropomorphizing the AI, attributing more human-like qualities like benevolence. For a more detailed interpretation of these findings, as well as discussion on practical implications and ethical considerations for AI design, please see the Supplementary Materials, where we elaborate on how a reliability-based approach may be more ethically sound and less misleading, and how these insights can guide developers and policymakers in their communication strategies.

### Limitations

This study has several limitations that need to be acknowledged. First, the observed increase in benevolence could be attributed to a framing effect due to the specific wording in the definition of trustworthy AI provided to the participants in the grouptrust condition (see Supplementary Materials for more details). The description highlighted that trustworthy AI “should care about ethical norms and/or conceptualize moral principles”, which might have influenced participants’ perceptions. Given the complex nature of trust and the various philosophical debates surrounding its definition, future research will need to walk a difficult tightrope.

Second, while we observed that increased perceived usefulness and benevolence did not lead to a corresponding increase in intention to use, contrary to what would have been expected (see^13–15^), this may be due to other factors unrelated to these variables. For instance, it is possible that participants had already formed opinions about whether they would use this technology, which were not influenced by the labels provided in the study. Future research could address this by employing a within-subject design to assess whether changing the label impacts participants’ perceptions over the course of the study.

Third, this study only measured user perceptions toward *automotive* AI. Future research should consider the effect of the trustworthy label on other AI technologies that are less tool-like, such as care-bots and chatbots.

### Conclusion

Labeling AI as “trustworthy” rather than “reliable” does not significantly impact user evaluations or acceptance, except increasing the likelihood that users will perceive AI as caring about our wellbeing. The findings on the lack of effect on users should be distinguished from the philosophical and ethical arguments on the lack of justification for the attribution of trust to AI. If emphasizing reliability over trustworthiness is more practical and ethically sound in the development and deployment of AI, the findings suggest that there is no cost to do so in future policies and developer practices in the AI industry.

## Methods

### Participants

Initially, 617 participants were recruited for the study through online platforms Cloud Research and Amazon Mechanical Turk. Recruitment began on June 24th, 2024 and completed on June 28th, 2024. Participants were paid approximately $12 an hour for completing the study, whether their data was ultimately analyzed or not. Before beginning with the study, participants gave their informed written consent using the online form. This study, including all experimental protocols, was approved by the Ethics Committee of the Faculty of Philosophy, Philosophy of Science, and Religious Studies at Ludwig Maximilian University of Munich. All experiments were conducted in accordance with relevant guidelines and regulations governing research with human participants.

After conducting a language proficiency check, 66 participants were excluded, leaving 551 (see Supplementary Materials). An additional 58 participants were excluded due to failing the attention check, resulting in 493 participants (see Supplementary Materials). A further 15 participants were excluded based on age criteria, leaving a final sample of 478 participants whose data were analyzed. The majority of participants were either male (246) or female (242). There were also 3 participants who identified as non-binary, 1 participant who preferred not to say, and 1 who identified as ‘other’ (for more, see Supplementary Materials).

As outlined in the pre-registered plan, the sample size was determined through a power analysis, ensuring sufficient statistical power to detect meaningful effects in the primary dependent variables. To account for potential exclusions due to language and attention check failures, an additional 10% was added to the calculated sample size, leading to a planned total of 440 participants. For the variables corresponding to the vignettes (Hypotheses H1 to H4), the power analysis was conducted using analytic methods based on effect sizes calculated from Cohen’s d, derived from the pilot study data on 5-point Likert scale variables. The analysis indicated that a sample size of 400 participants would yield sufficient power $(0.80)$ to detect the observed effect sizes at a significance level of $\alpha = 0.05$.

For the TAM questionnaire (H5 and H5.1–H5.8), the effect sizes observed from the pilot study for the 5-point Likert scale variables were $-0.193$, $-0.257$, $-0.507$, and $-0.431$. These were used alongside threshold estimates and their standard errors, which were $4.8725$ $(\text {SE} = 0.5113)$, $3.5229$ $(\text {SE} = 0.4330)$, $1.2539$ $(\text {SE} = 0.3788)$, and $-1.7388$ $(\text {SE} = 0.3885)$, respectively. The standard deviations of participants and items were $1.4363$ and $0.3499$, respectively. With these parameters, a sample size of 400 participants was determined to provide a power of $0.80$ at a significance level of $\alpha = 0.05$. This was confirmed through simulation involving 100 iterations, each with 400 participants, ensuring robustness in the power estimate.

During data collection, an initial passing sample of 390 participants revealed a gender imbalance, with approximately 65% male and 35% female participants (245 male participants and 145 female participants). To achieve a balanced gender demographic as outlined in pre-registration, additional data were collected exclusively from women, resulting in a more balanced sample. Our preliminary analysis indicated that gender, specifically being female, significantly influenced attitudes toward automotive AI, with women reporting more negative attitudes. This finding underscored the importance of ensuring a balanced sample to accurately capture the diversity of perspectives (for more detailed information, see Supplementary Materials).

We categorized participants into three age groups for analysis: 18-30, 30-45, and 45-65. Due to the nature of these categories, there is potential ambiguity at the boundaries, specifically for participants aged exactly 30 and 45. In our dataset, we could not precisely allocate these boundary ages to a specific group. Consequently, there may be classification ambiguity at these boundaries, which could influence the interpretation of age-related findings (for more, see Supplementary Materials).

### Experimental design

A one-way between-subjects design was used. Participants were randomly assigned to one of two groups: the “trustworthy AI” group or the “reliable AI” group. The assignment was random to ensure that each participant had an equal chance of being placed in either group. Ultimately, data were analyzing from 241 participants assigned to the “trustworthy AI” group and 237 participants assigned to the “reliable AI” group, ensuring a balanced sample. The sample consisted of 478 participants, with a balanced distribution in terms of gender and age groups. After group assignment, participants progressed through 4 phases.

#### Phase 1: Demographic phase

This section involved the collection of demographic data (age, gender, education, experience with automotive AI, driver’s license status, AI expertise), and a language check to ensure fluency in English. Detailed demographic data and language check criteria are described in Supplementary Materials.

#### Phase 2: Induction phase

Participants were presented with group-specific definitions of “trustworthy AI” or “reliable AI” (see Supplementary Materials for the language check criteria, as well as an attention check to ensure participation and comprehension of the respective definition). Similarly to concept-induction methods used in free will studies (see, e.g., Vohs and Schooler^23^), these brief textual descriptions were intended to promote the conceptual distinction between “trustworthy” and “reliable”.

#### Phase 3: Vignette phase

Participants read three separate vignettes, one for each automotive AI-assisted task: planning assistance, parking assistance, and steering assistance. The vignettes were presented in random order and, across the two groups, the vignettes were identical except for the key terms “trustworthy” or “reliable” (and other related semantic derivatives) respective of group assignment. Participants answered the same four questions for each of the three vignettes (AI accountability, AI blameworthiness, confidence in driving, and confidence in learning) using a 5-point Likert scale, which were also presented in random order. The full text of the vignettes and the questions asked are available in Supplementary Materials.

#### Phase 4: Questionnaire phase

Participants answered the eight TAM questions covering the distinct constructs perceived ease of use, perceived usefulness, behavioral intention, ability trust, human-like trust (benevolence and integrity), general trust, and attitude. A 5-point Likert scale was used to measure responses. The questions were presented in random order to prevent biasing effects. The complete TAM questionnaire is provided in Supplementary Materials. As these measures reflect the general perceptions of AI by participants, comparing responses between the “trustworthy AI” and “reliable AI” groups serves as an indirect manipulation check, an approach that aligns with previous research demonstrating that brief textual inductions can shape conceptual frameworks, as seen in the free will studies mentioned above.

The selection of items for TAM is informed by Choung et al.^15^, which identified the importance of trust (general, human-like, and ability-based trust) in AI and its impact on technology acceptance. Their comprehensive study provides a validated framework for measuring key constructs related to technology acceptance. Each TAM variable category was measured with one question to streamline the data collection process, minimize participant fatigue, and cohere with automotive AI.

The selected questions are the most appropriate for automotive AI, and the wording of each question has been modified to reflect this technology. The original study asked about smart AI assistants and, as a result, several of the survey items do not apply. The human-like trust variable is assessed using two questions as in the original study in order to ensure a comprehensive and reliable measurement. This is necessary due to the complexity and multidimensional nature of human-like trust, which includes two distinct elements, benevolence and integrity.

### Data collection and handling

Data was collected using the Qualtrics online survey platform. Procedures for data handling and storage included secure, encrypted storage and removal of personal identifiers. Data monitoring and quality assurance were ensured through exclusion of incomplete responses, failed language checks, and failed attention checks. See Supplementary Materials.

### Data analysis

For the analysis of the vignettes, independent samples t-tests were conducted to compare means by condition for hypotheses related to the vignette assessments, i.e. H1 to H4, which include AI accountability, AI blameworthiness, confidence in using automotive AI, and confidence in learning how to drive with automotive AI (in the context of obtaining one’s first driver’s license). To further investigate the effects of condition on outcomes related to the vignettes, Bayesian ordinal regression models were fitted for each question.

For the total TAM score, H5, we employed a Cumulative Link Mixed Model (CLMM) to account for the ordinal nature of the response variable and to model participant and TAM items as random effects. Cumulative Link Models (CLMs) were used to analyze ordinal response variables related to the individual TAM questions, specifically hypotheses H5.1 through H5.8.

Analysis deviated slightly from the pre-registered plan. First, the residuals of the t-test did not adhere to a normal distribution, as indicated by significant deviations in the Shapiro-Wilk test results. Since the t-test assumes normally distributed residuals, its application was questioned for this data. We still performed the t-test and report the results below. To address the non-normality of residuals, we also conducted a Wilcoxon test as a non-parametric alternative; however, this test did not reveal any significant results either. Detailed results of the Wilcoxon test are reported in Supplementary Materials. Finally, we opted not to use Cumulative Link Mixed Models for H5.1 through H5.8 as initially proposed. Since we modeled each question separately, we did not need to account for random effects of items or participants, so a simpler ordinal regression model (CLM) was sufficient for analysis.

## Supplementary Information

Supplementary Information.

## Data Availability

All data supporting the findings of this study are available in the Open Science Framework repository at https://osf.io/6un4h/ with the DOI 10.17605/OSF.IO/6UN4H. The repository includes all relevant datasets and the code used for analysis. There are no restrictions on data availability, and the materials are freely accessible for non-commercial use.
