# Supplementary material for: The impact of labeling automotive AI as trustworthy or reliable on user evaluation and technology acceptance
Source: Sci Rep. 2025 Jan 9;15:1481. doi: 10.1038/s41598-025-85558-2 (PMC11718277; doi:10.1038/s41598-025-85558-2)
Supplement: Supplementary file 1 — Supplementary Information. [file 41598_2025_85558_MOESM1_ESM.pdf]

# The impact of labeling automotive AI as trustworthy or reliable on user evaluation and technology acceptance

John Dorsch[1, 4*], Ophelia Deroy[1, 2, 3]

**1** Faculty of Philosophy, Philosophy of Science and the Study of Religion, Ludwig-Maximilians-Universität München, Munich, Germany
**2** Munich Center for Neurosciences, Ludwig-Maximilians-Universität München, Munich, Germany
**3** Institute of Philosophy, School of Advanced Study, University of London, London, UK
**4** Institute of Philosophy, Czech Academy of the Sciences, Prague, Czech Republic

* johndorsch@gmail.com

## Supplementary Material

### Extended Model Diagnostics and Statistical Details

#### Bayesian ordinal regression for Vignettes

To further examine the potential differences between the "Reliable AI" and "Trustworthy AI" conditions, Bayesian ordinal regression models were applied to each of the four questions in the vignette study (see Table 1). The analysis yielded small estimated group effects across all questions, with 95% credible intervals consistently encompassing zero, indicating no strong evidence for significant differences between the groups. Specifically, the probabilities that the group effect is greater than zero were all below 0.5, further underscoring the absence of a positive effect. The Bayesian models demonstrated good convergence, with Rhat values close to 1.00 and sufficient effective sample sizes, reinforcing the reliability of the estimates. Overall, these results corroborate the earlier findings from the traditional t-tests and Wilcoxon tests, suggesting that any observed differences are minimal or non-existent.

**Table 1.** Summary of Bayesian Analysis Results for Vignette Questions

| Main Question | Estimate | 95% CI (Lower) | 95% CI (Upper) | Probability (Effect > 0) |
|---|---|---|---|---|
| AI Accountability | -0.2175 | -0.8834 | 0.4387 | 0.26425 |
| AI Blameworthiness | -0.0296 | -0.6699 | 0.6185 | 0.46325 |
| Confidence in Driving | -0.3043 | -0.8843 | 0.2742 | 0.15650 |
| Confidence in Learning | -0.2941 | -0.9500 | 0.3188 | 0.17950 |

#### H5: Total TAM Score

For the Total TAM Score, we conducted ordinal regression analyses to examine the relationship between the condition and participants' perceptions across the total sum of the various questions. The mixed model had a log-likelihood of $-4498.18$ and an AIC of 9010.35. The model converged after 538 iterations, with a maximum gradient of

$1.01 \times 10^{-3}$ and a condition number of $1.8 \times 10^{02}$. The coefficient for the `grouptrust` variable was estimated at 0.2058 with a standard error of 0.2137, resulting in a $z$-value of 0.963 and a $p$-value of 0.335. The 95% confidence interval for this estimate ranged from $-0.2131$ to 0.6247, indicating no statistically significant effect of the "trustworthy AI" label on the Total TAM Score. The thresholds were estimated as follows: Strongly Disagree|Disagree at $-4.0489$ (CI: $[-4.5804, -3.5173]$), Disagree|Neutral at $-2.2495$ (CI: $[-2.7636, -1.7354]$), Neutral|Agree at 0.0009 (CI: $[-0.5069, 0.5086]$), and Agree|Strongly Agree at 3.4428 (CI: $[2.9192, 3.9664]$). None of the threshold differences reached statistical significance. The Brant test for the parallel regression assumption of the Cumulative Link Mixed Model (CLMM) indicated that the assumption holds, with an omnibus test result of $\chi^2(3) = 7.11$, $p = 0.07$. Specifically, the test for the `grouptrust` variable also yielded $\chi^2(3) = 7.11$, $p = 0.07$.

### H5.1 - H5.8: Results of Individual Technology Acceptance Items

For individual TAM items, we conducted ordinal regression analyses to examine the relationship between the group label and participants' perceptions across the various questions: 1. perceived ease of use, 2. perceived usefulness, 3. intention to use, 4. trust: ability, 5. trust: benevolence, 6. trust: integrity, 7. trust: general, 8. attitude general. As outlined in our pre-registration, we tested each question for adherence to the proportional odds assumption using the Brant test, implemented through the `brant` package in R. The results indicated that Question 4 (trust in ability) did not satisfy this assumption ($\chi^2 = 8.65$, $df = 3$, $p = 0.0343$). To maintain consistency across our analyses and ensure a uniform approach for all eight questions, we opted to use a model with flexible thresholds. This approach allowed us to account for potential variability in the relationship between the labels and responses across different thresholds. Each model included 478 observations, using a logit link function with flexible thresholds. Below are the detailed results for each question, including the specific model fit statistics, coefficients, and threshold estimates with their respective confidence intervals.

### Question 1: Learning to use automotive AI would be easy for me

For Question 1, the model had a log-likelihood of $-630.88$ and an AIC of 1271.77. The model converged after six iterations, with a maximum gradient of $3.92 \times 10^{-13}$ and a condition number of $2.5 \times 10^{01}$. The coefficient for the `grouptrust` variable was estimated at 0.3354 with a standard error of 0.1697, resulting in a $z$-value of 1.976 and a $p$-value of 0.0481. The 95% confidence interval for this estimate ranged from 0.0028 to 0.6679, indicating a statistically significant effect of the "trustworthy AI" label on the perception of ease of learning. The thresholds were estimated as follows: Strongly Disagree|Disagree at $-3.6908$ (CI: $[-4.3346, -3.0470]$), Disagree|Neutral at $-1.7084$ (CI: $[-2.0130, -1.4038]$), Neutral|Agree at $-0.3899$ (CI: $[-0.6386, -0.1412]$), and Agree|Strongly Agree at 1.7564 (CI: $[1.4553, 2.0575]$). The result for the Neutral|Agree threshold difference was significant ($p = 0.016$), with a standard error of 0.1391 and a difference of 0.4624. The other threshold differences did not reach significance.

### Question 2: Using automotive AI would improve my performance at accomplishing driving-related tasks

For Question 2, the model had a log-likelihood of $-688.55$ and an AIC of 1387.09. The model converged after five iterations, with a maximum gradient of $2.10 \times 10^{-08}$ and a condition number of $1.9 \times 10^{01}$. The coefficient for the `grouptrust` variable was estimated at $-0.0318$ with a standard error of 0.1657, resulting in a $z$-value of $-0.192$ and a $p$-value of 0.848. The 95% confidence interval for this estimate ranged from

−0.3566 to 0.2929, indicating that the effect of the "trustworthy AI" label on performance improvement is not statistically significant. The thresholds were estimated as follows: Strongly Disagree|Disagree at −2.5237 (CI: $[-2.9003, -2.1470]$), Disagree|Neutral at −1.3710 (CI: $[-1.6465, -1.0956]$), Neutral|Agree at −0.5171 (CI: $[-0.7691, -0.2652]$), and Agree|Strongly Agree at 1.5071 (CI: $[1.2077, 1.8065]$). None of the thresholds reached statistical significance.

**Question 3: Using automotive AI is something I would do in the future or will continue to do so.**

For Question 3, we examined how the group labels ("trustworthy AI" vs. "reliable AI") relate to participants' intention to use the technology. We used an ordinal regression model with a logit link and flexible thresholds. The model included 478 observations, had a log-likelihood of −704.85, and an AIC of 1419.70. It converged after five iterations, with a maximum gradient of $7.07 \times 10^{-10}$ and a condition number of $2.2 \times 10^{01}$.

The coefficient for the `grouptrust` variable was −0.0877 (SE = 0.1653, $z = -0.531$, $p = 0.596$), and the 95% confidence interval ranged from −0.4116 to 0.2363. This indicates no statistically significant effect of the "trustworthy AI" label on the intention to use the technology.

Threshold coefficients mark where responses shift between categories on the Likert scale. The threshold between "Strongly Disagree" and "Disagree" was −2.4660 (SE = 0.1878, 95% CI $[-2.8340, -2.0981]$). Between "Disagree" and "Neutral" it was −1.3005 (SE = 0.1393, 95% CI $[-1.5735, -1.0275]$). Between "Neutral" and "Agree" it was −0.2716 (SE = 0.1249, 95% CI $[-0.5163, -0.0268]$). Finally, between "Agree" and "Strongly Agree" it was 1.6065 (SE = 0.1489, 95% CI $[1.3147, 1.8984]$). Overall, these results suggest that the "trustworthy AI" label does not significantly influence participants' future intentions to use automotive AI.

**Question 4: Automotive AI technologies are competent in their area of expertise**

For Question 4, the model had a log-likelihood of −622.62 and an AIC of 1255.24. The model converged after six iterations, with a maximum gradient of $3.13 \times 10^{-13}$ and a condition number of $1.9 \times 10^{01}$. The coefficient for the `grouptrust` variable was estimated at −0.0442 with a standard error of 0.1698, resulting in a $z$-value of −0.26 and a $p$-value of 0.795. The 95% confidence interval for this estimate ranged from −0.3771 to 0.2886, indicating that the effect of the "trustworthy AI" label on the perception of competence is not statistically significant. The thresholds were estimated as follows: Strongly Disagree|Disagree at −3.2074 (CI: $[-3.6975, -2.7173]$), Disagree|Neutral at −1.8894 (CI: $[-2.2027, -1.5762]$), Neutral|Agree at −0.3087 (CI: $[-0.5557, -0.0618]$), and Agree|Strongly Agree at 2.1247 (CI: $[1.7871, 2.4623]$).

**Question 5: Automotive AI technologies care about our well-being**

For Question 5, the model had a log-likelihood of −714.40 and an AIC of 1438.81. The model converged after five iterations, with a maximum gradient of $8.39 \times 10^{-09}$ and a condition number of $2.4 \times 10^{01}$. The coefficient for the `grouptrust` variable was estimated at 0.4862 with a standard error of 0.1651, resulting in a $z$-value of 2.945 and a $p$-value of 0.00323. The 95% confidence interval for this estimate ranged from 0.1626 to 0.8097, indicating a statistically significant positive effect of the "trustworthy AI" label on the perception that automotive AI technologies care about our well-being. The thresholds were estimated as follows: Strongly Disagree|Disagree at −1.2419 (CI: $[-1.5177, -0.9662]$), Disagree|Neutral at −0.3804 (CI: $[-0.6267, -0.1341]$),

Neutral|Agree at 1.0483 (CI: $[0.7878, 1.3087]$), and Agree|Strongly Agree at 2.8723 (CI: $[2.4721, 3.2725]$). The results for the Strongly Disagree|Disagree threshold difference was significant ($p = 0.0013$), with a standard error of 0.1924 and a difference of 0.7862. The result for the Neutral|Agree threshold difference was also significant ($p = 0.011$), with a standard error of 0.1338 and a difference of 0.5065. The other threshold differences did not reach significance.

### Question 6: Automotive AI technologies do not abuse the information and advantage they have over their users

For Question 6, the model had a log-likelihood of $-666.32$ and an AIC of 1342.65. The model converged after five iterations, with a maximum gradient of $1.14 \times 10^{-07}$ and a condition number of $1.6 \times 10^{01}$. The coefficient for the `grouptrust` variable was estimated at 0.1857 with a standard error of 0.1671, resulting in a $z$-value of 1.111 and a $p$-value of 0.267. The 95% confidence interval for this estimate ranged from $-0.1419$ to 0.5132, indicating that the effect of the "trustworthy AI" label on the perception of information abuse is not statistically significant. The thresholds were estimated as follows: Strongly Disagree|Disagree at $-2.3873$ (CI: $[-2.7580, -2.0166]$), Disagree|Neutral at $-1.3978$ (CI: $[-1.6782, -1.1173]$), Neutral|Agree at 0.4396 (CI: $[0.1940, 0.6852]$), and Agree|Strongly Agree at 2.3124 (CI: $[1.9653, 2.6596]$). None of the thresholds reached statistical significance.

### Question 7: I trust that automotive AI can offer information and service that is in my best interest

For Question 7, the model had a log-likelihood of $-648.60$ and an AIC of 1307.19. The model converged after six iterations, with a maximum gradient of $5.36 \times 10^{-14}$ and a condition number of $2.0 \times 10^{01}$. The coefficient for the `grouptrust` variable was estimated at 0.1035 with a standard error of 0.1698, resulting in a $z$-value of 0.61 and a $p$-value of 0.542. The 95% confidence interval for this estimate ranged from $-0.2292$ to 0.4362, indicating that the effect of the "trustworthy AI" label on the perception that automotive AI offers information and service in the user's best interest is not statistically significant, and none of the thresholds reached statistical significance.

### Question 8: I feel positive toward automotive AI technologies

For Question 8, the model had a log-likelihood of $-691.06$ and an AIC of 1392.11. The model converged after five iterations, with a maximum gradient of $1.10 \times 10^{-07}$ and a condition number of $2.4 \times 10^{01}$. The coefficient for the `grouptrust` variable was estimated at $-0.0022$ with a standard error of 0.1663, resulting in a $z$-value of $-0.013$ and a $p$-value of 0.989. The 95% confidence interval for this estimate ranged from $-0.3282$ to 0.3238, indicating that the effect of the "trustworthy AI" label on the positive perception of automotive AI technologies is not statistically significant. No threshold differences reached significance.

## Goodness of Fit Evaluation

### T-Test Analysis and Further Analysis: Vignette Questions

The Shapiro-Wilk test results for the residuals revealed significant deviations from normality across all questions. For "AI Accountability," the Shapiro-Wilk statistic was 0.898 with a $p$-value of $8.42 \times 10^{-30}$. Similarly, "AI Blameworthiness" had a statistic of 0.890 and a $p$-value of $9.94 \times 10^{-31}$, and "Confidence in Driving" showed a statistic of 0.890 with a $p$-value of $8.59 \times 10^{-31}$. For "Confidence in Learning," the Shapiro-Wilk

statistic was 0.884 with a $p$-value of $1.70 \times 10^{-31}$. These results consistently demonstrate that the residuals significantly deviate from a normal distribution, which invalidates the assumptions underlying the t-tests that were initially planned for the analysis.

Given the violation of normality assumptions in the residuals, the Wilcoxon test was employed as a non-parametric alternative to assess differences between the groups. This test is particularly suitable because it does not rely on the assumption of normality and is robust to deviations from this assumption. The results of the Mann-Whitney U tests showed no significant differences between the groups for any of the main questions. For "AI Accountability," the Wilcoxon statistic was 265,040 with a $p$-value of 0.286. "AI Blameworthiness" had a Wilcoxon statistic of $258,296$ and a $p$-value of 0.866. For "Confidence in Driving," the statistic was $265,950$ with a $p$-value of 0.229, and for "Confidence in Learning," the statistic was 262,538 with a $p$-value of 0.460. These results confirm that, even when using a test suited for non-normal data, there are no significant differences between the groups for the questions examined.

### CLMM Analysis: Total TAM Score

Given that the Cumulative Link Mixed Model does not assume a normal distribution of residuals, we assessed the goodness of fit using likelihood ratio tests instead of residual analysis. The likelihood ratio test compares the fit of a null model, which includes only the random effects, to a full model that also includes the group effect. The results of this test indicate that the inclusion of the group variable does not significantly improve the model fit. Specifically, the likelihood ratio statistic was 0.9269 with a $p$-value of 0.3357, suggesting that the model's fit does not improve significantly when the group variable is included. This supports the finding that the group effect is not statistically significant, as reflected in the earlier analysis. The non-significant $p$-value further confirms that the inclusion of the group variable does not contribute meaningfully to explaining the variability in the response.

### CLM Analysis: Individual TAM Questions

For the same reason as above, we have evaluated the goodness of fit of Cumulative Link Models through likelihood ratio tests, instead of residual analysis as originally planned. These tests compare the fit of the base model, which includes only the group variable, to models incorporating additional predictors. For the base model in each TAM question, the likelihood ratio test results reveal that, in all but one case, adding additional predictors does not significantly improve model fit. Specifically, $p$-values for Questions 1, 2, 3, 6, 7, and 8 range from 0.245 to 0.980, indicating that the base model performs comparably to models with added predictors. The exception is Question 4, where a $p$-value of 0.029 suggests a marginal improvement in model fit with additional predictors. Thus, overall, the base model, which includes only the group variable, appears to be a generally adequate fit for the data across questions. For example, none of the models showed a significant improvement in fit when including gender as a predictor and its interaction with group ($p$-values ranged from 0.387 to 0.896), except for Question 6 where the $p$-value was 0.013, indicating an improvement. However, this did not affect the significance of the findings. For instance, the $p$-value for the coefficient of `grouptrust` is 0.757, for `Female` is 0.163, and for the interaction term `grouptrust` is 0.426.

## Outlier Analysis

We conducted an outlier analysis using the Interquartile Range (IQR) method to identify extreme values across each TAM question. Specifically, outliers were defined as

observations that fell below the first quartile (Q1) minus 1.5× the IQR or above the third quartile (Q3) plus 1.5× the IQR.

Across all eight questions, the total number of responses was consistent at 478, with the majority of outliers selecting “Strongly Disagree”, the one exception being Question 5, which had no outliers. For Question 5, the median response was “Agree,” with an interquartile range (IQR) from “Neutral” to “Agree.”

For Question 1, there were 10 outliers, with a median of “Neutral” and an IQR from “Disagree” to “Agree.” Question 2 had 36 outliers, with a median of Neutral” and an IQR of Disagree” to “Agree.” In Question 3, 39 outliers were identified, with a median of Disagree” and an IQR from “Disagree” to “Agree.” Question 4 showed 19 outliers, with a median of “Disagree” and an IQR from “Disagree” to “Agree.” Question 6 had 37 outliers, with a median of Neutral” and an IQR from “Disagree” to “Agree.” For Question 7, 29 outliers were noted, with a median of Disagree” and an IQR from “Disagree” to “Agree.” Finally, Question 8 had 36 outliers, with a median of “Neutral” and an IQR from “Disagree” to “Agree.”

Consistent with our pre-registered plan, these outliers were not excluded from analysis. The rationale for retaining outliers is based on the premise that they may reflect genuine variability in participant responses. Excluding these data points could potentially bias our results and lead to an underestimation of the true variability within the sample. Therefore, all identified outliers were included in the final analyses to ensure a comprehensive representation of participant behavior and perceptions. In our analysis, the presence of outliers did not significantly distort the model estimates or alter the interpretation of the results. This supports our decision to retain these data points, as they provide valuable insights into the behavior and perceptions of individuals at the extremes of the distribution.

## Bayesian Analysis of Vignette Questions

### Model Summary

The Bayesian cumulative link mixed model (CLMM) was fitted using the following settings:

```
Family: cumulative
Links: mu = logit; disc = identity
Formula: response ~ group + (1 | participant) + (1 | vignette)
Data: current_data (Number of observations: 1434)
Draws: 4 chains, each with iter = 4000; warmup = 1000; thin = 1;
total post-warmup draws = 12000
```

### Multilevel Hyperparameters

The multilevel (random effects) parameters for participants and vignettes are as follows:

```
~participant (Number of levels: 478)
Estimate    Est.Error    l-95% CI    u-95% CI    Rhat    Bulk_ESS    Tail_ESS
3.31        0.18         2.96        3.68        1.00    2740        4853

~vignette (Number of levels: 3)
Estimate    Est.Error    l-95% CI    u-95% CI    Rhat    Bulk_ESS    Tail_ESS
0.53        0.55         0.08        2.06        1.00    2942        4237
```

### Regression Coefficients

The regression coefficients for the model, including the intercepts and the group effect, are provided below:

```
Regression Coefficients:
Estimate       Estimate   Est. Error  l-95% CI    u-95% CI   Rhat    Bulk_ESS    Tail_ESS
```

| | | | | | | | |
|---|---|---|---|---|---|---|---|
| Intercept[1] | -5.71 | 0.49 | -6.65 | -4.67 | 1.00 | 2586 | 3749 |
| Intercept[2] | -3.23 | 0.45 | -4.09 | -2.26 | 1.00 | 2239 | 3712 |
| Intercept[3] | -1.14 | 0.44 | -1.96 | -0.18 | 1.00 | 2273 | 3740 |
| Intercept[4] | 3.13 | 0.45 | 2.33 | 4.12 | 1.00 | 2710 | 3530 |
| groupt | -0.29 | 0.32 | -0.95 | 0.32 | 1.00 | 1741 | 3197 |

### Conclusion

The Bayesian analysis results indicate that there is no strong evidence of a significant difference between the "Reliable AI" and "Trustworthy AI" groups across the main questions. The group effect estimates were close to zero, with the 95% credible intervals including zero, and the probabilities that the group effect is greater than zero were consistently below 0.5.

### Descriptive Statistics

The demographic characteristics of the sample are presented below. The sample consisted of 493 participants with a balanced distribution in terms of gender and age groups. 15 participants were excluded from analysis due to being 65 years old or older, leaving a final sample of 478 participants whose data were analyzed. The majority of participants were either male (246) or female (242). There were also 3 participants who identified as non-binary, 1 participant who preferred not to say, and 1 who identified as 'other'. The age distribution was as follows: 68 participants were 18-30 years old, 240 were 30-45 years old, 167 were 45-64 years old, 15 were 65 years or older, and 3 preferred not to say. A vast majority of participants held a driver's license (476), while 14 participants did not, and 3 preferred not to disclose their status.

The participants reported various areas of expertise, including 9 in philosophy, 32 in computer science, 5 in informatics, 8 in data science, 10 in computer programming, 3 in machine learning, and 1 in robotics, with no participants selecting 'other'. The educational background of participants varied widely: 212 had a bachelor's degree (4-year), 53 had an associate degree (2-year), 52 had a master's degree, 81 had some college but no degree, 8 had a professional degree (JD, MD), 68 had completed high school (including GED), 3 had less than high school education, 8 had technical certification, 7 had a doctoral degree, and 1 preferred not to say. No participants selected 'other' for education. Participants had various experiences with autonomous technology, with 221 participants reporting some experience with automotive AI, such as with lane-keeping, steering, route-planning, or parking assistance (see Experience for the specific questions).

## Definitions of Key Terms

Participants were instructed to read and understand the definitions of "trustworthy" and "reliable" AI, as appropriate to their group assignment. These definitions were also reiterated when participants read the vignettes. Definitions were developed and informed by normative analysis and ethical considerations in previous work in the ethics of AI [3].

### Trustworthy AI

1. It should be lawful, complying with all applicable laws and regulations.
2. It should be ethical and do the right thing for the right ethical reasons.
3. It should care about ethical norms and/or conceptualize moral principles.

### Reliable AI

1. It should be lawful, complying with all applicable laws and regulations.
2. It should be effective, shown to produce the expected outcome for the tasks it was assigned.
3. It should be consistent, likely to produce such outcomes in the future.

## Inclusion Criteria

To be considered for inclusion in the study, participants were required to demonstrate a minimum threshold of fluency in English. Specifically, only those achieving a success rate of at least 75% on the language check were included in the final analysis. Additionally, participants were required to meet a minimum accuracy threshold of 75% on the attention check to ensure comprehension of the key components, one that was specific to group assignment.

### Language Check

Please select **all** the sentences below that are written in **correct English**:

1. Atheletes often need to warm up.
2. I just saw a moose running down the road!
3. John ill felt and went doctor.
4. Could you the books put in that boxes?
5. I am forget do my homeworks.
6. She think English is more easier to learn.
7. Tommorrow was tatiehr than today.
8. The building is a very murnnlye.
9. Where's the pen I gave you yesterday?
10. He was pulled over by the police for driving 120 miles per hour.

## Attention Check

These attention checks were shown below the corresponding definitions to ensure participants read how the terms "trustworthy AI" and "reliable AI", respectively, were meant to be understood (for the definitions, see Definitions of Key Terms).

### Trust Group

**Before continuing, please select only the statements below that align with the key components of trustworthy AI.**

1. If the AI is trustworthy, it will do the right thing for the right ethical reasons.
2. If the AI is trustworthy, it neither conceptualizes moral principles nor does it care about ethical norms.
3. Trustworthy AI cares about ethical norms and/or conceptualizes moral principles.
4. Trustworthy AI has nothing to do with the AI having moral principles or ethical norms.

### Reliable Group

**Before continuing, please select only the statements below that align with the key components of reliable AI.**

1. If the AI is reliable, it will produce the expected outcome for the tasks it was assigned.
2. If the AI is reliable, it will neither produce the expected outcome, nor is it likely to do so in the future.
3. Reliable AI consistently produces the expected outcome.
4. Reliable AI has nothing to do with the AI being effective or consistent.

## Vignettes

Participants were instructed to read three distinct vignettes related to automotive AI: Planning Assistance, Parking Assistance, and Steering Assistance. Each vignette was designed to evaluate AI accountability, AI blameworthiness, confidence in using the AI, and confidence in learning to drive. The vignettes were identical except for the key terms "trustworthy" and "reliable," as specified by the participants' group assignment. The key terms were not in bold as they are below, but italicized.

### Planning Assistance

Picture yourself in the driver's seat of a modern vehicle. You're stuck in the midst of heavy traffic during your daily commute. The vehicle is equipped with a **trustworthy/reliable** AI route-planning system, designed by the manufacturer to analyze real-time traffic data and recommend a new route to avoid congestion. You **trust/rely** on the suggestion, and the AI switches lanes and diverts to the suggested route without any additional input from you, the driver.

### Parking Assistance

Visualize yourself navigating a suburban neighborhood in your vehicle equipped with **trustworthy/reliable** AI parking assistance. You come across a tight parallel parking space, and the AI recommends activating the **trustworthy/reliable** AI parking assistance. You **trust/rely** on the AI to park the vehicle, and it maneuvers the car into the tight parking spot without any additional input from you, the driver.

### Steering Assistance

Imagine driving on a rural road with your car's **trustworthy/reliable** AI driving assistance providing real-time safety alerts. As you approach a curve, the **trustworthy/reliable** AI uses its sensors to detect an obstacle ahead and displays a warning on your dashboard screen. It also suggests that the AI should steer the vehicle to bypass the obstacle. You **trust/rely** on the suggestion, and the AI steers the vehicle to avoid the obstacle without any additional input from you, the driver.

## Experience

Participants were asked to check any of the following boxes that correspond to their experience with autonomous automotive technology. For the purpose of this study, 'autonomous' refers to the vehicle's ability to perform specific actions (such as planning

a route, maintaining lane position, parking the vehicle, or steering) independently of the driver. Checking any of these boxes indicated that the participant was assigned to the 'Experience' category.

- I have experience driving a vehicle with autonomous technology for lane-keeping assistance.
- I have experience driving a vehicle with autonomous technology for route-planning assistance.
- I have experience driving a vehicle with autonomous technology for parking assistance.
- I have experience driving a vehicle with autonomous technology for steering assistance.
- I have had an automobile accident while driving a vehicle equipped with one of the autonomous technologies listed above.

## Technology Acceptance Model Questionnaire

The items in the Technology Acceptance Model questionnaire were presented in a random order to prevent biasing effects. Both groups, "Trustworthy AI" and "Reliable AI," answered the questionnaire. The TAM questionnaire is based on Choung et al [14]. Titles of the constructs were not shown to participants.

1. **Perceived Ease of Use**: Learning to use automotive AI has been or would be easy for me.
2. **Perceived Usefulness**: Using automotive AI has improved or would improve my performance at accomplishing driving-related tasks.
3. **Intention to Use**: Using automotive AI is something I would do in the future or will continue to do so.
4. **Ability Trust**: Automotive AI technologies are competent in their area of expertise.
5. **Benevolence Trust**: Automotive AI technologies care about our well-being.
6. **Integrity Trust**: Automotive AI technologies do not abuse the information and advantage they have over their users.
7. **General Trust**: I trust that automotive AI can offer information and services that are in my best interest.
8. **Attitude**: I feel positive toward automotive AI technologies.

## Supplementary Analysis

Additional analyses were conducted to explore the impact of demographic variables, such as gender, age, and experience with AI, on the main outcomes. These analyses were considered exploratory, as specified in the pre-registration document (Section 8.1), which states that any analyses not part of the primary hypotheses but providing additional insights would be exploratory. Specifically, we examined interactions between demographic variables (gender, age, and experience) and the dependent variables. An ordinal regression model was employed to assess the influence of demographic factors.

The model was fit using the `clmm` function, which allows for the inclusion of both fixed and random effects. The model specification was as follows: `ordinal_model <- clmm(response ~ demo_variable + (1 | participant), data = current_data)`.

## Gender

In this analysis, we examined the impact of gender on various attitudes towards automotive AI technologies. Given the sparsity of data in certain gender categories, "Non-Binary," "Other," and "Prefer not to say" were combined into a single "Other" category to ensure more stable estimates. We utilized ordinal regression models to assess the relationship between gender and respondents' perceptions across the various questions asked above, the eight TAM questions and the four vignette questions. The models included 478 observations, and we report the coefficients and confidence intervals for the predictor variables. Below are the detailed results for each question, including the specific model fit statistics, coefficients, and confidence intervals for the predictor coefficients.

### AI Accountability

For AI Accountability, being female was associated with a significant decrease in AI accountability ratings (Estimate = $-0.836$, SE = 0.253, $z = -3.31$, $p = 0.000930$, CI = $[-1.33, -0.341]$). The "Other" category did not show a significant effect (Estimate = $-1.60$, SE = 1.66, $z = -0.962$, $p = 0.336$, CI = $[-4.85, 1.66]$).

### AI Blameworthiness

For AI Blameworthiness, females reported lower blameworthiness ratings (Estimate = $-0.734$, SE = 0.234, $z = -3.14$, $p = 0.0017$, CI = $[-1.19, -0.275]$). The "Other" category did not show a significant effect (Estimate = 1.53, SE = 1.61, $z = 0.951$, $p = 0.341$, CI = $[-1.62, 4.68]$).

### Confidence in Driving

For Confidence in Driving, there was a significant decrease in confidence in driving for females (Estimate = $-0.785$, SE = 0.290, $z = -2.71$, $p = 0.0067$, CI = $[-1.35, -0.217]$). The "Other" category showed a marginally significant effect (Estimate = $-2.40$, SE = 1.41, $z = -1.71$, $p = 0.088$, CI = $[-5.16, 0.357]$).

### Confidence in Learning

For Confidence in Learning, confidence in learning was significantly lower for females (Estimate = $-0.932$, SE = 0.242, $z = -3.85$, $p = 0.000117$, CI = $[-1.41, -0.458]$). The "Other" category also showed a significant negative effect (Estimate = $-3.41$, SE = 1.54, $z = -2.21$, $p = 0.027$, CI = $[-6.44, -0.383]$).

In summary, the analyses indicate that being female significantly negatively affects perceptions in several areas, including AI accountability, AI blameworthiness, confidence in driving, and confidence in learning. The combined "Other" category also showed significant negative effects in some areas, though often with larger confidence intervals, reflecting greater variability due to the lower number of observations in these groups.

### Technology Acceptance Questions

These results do not include Question 3 "Using automotive AI is something I would do in the future or will continue to do so" as this is discussed above under Intention to Use.

#### Question 1: Learning to use automotive AI would be easy for me

The model had a log-likelihood of $-619.43$ and an AIC of 1250.86. The coefficient for the `Female` variable was estimated at $-0.8482$ with a standard error of 0.1749, resulting in a $z$-value of $-4.849$ and a $p$-value of $1.24 \times 10^{-6}$, indicating a statistically significant negative effect of being female on the perception of ease of learning. The coefficient for the `Other` variable was estimated at $-1.6893$ with a standard error of 0.7398, resulting in a $z$-value of $-2.283$ and a $p$-value of 0.0224, indicating a statistically significant negative effect for the combined "Other" category. The 95% confidence intervals for these estimates ranged from $-1.19$ to $-0.507$ for `Female` and from $-3.17$ to $-0.207$ for `Other`.

Additionally, for Question 1, the interaction effect between being female and having no experience with automotive AI was found to be significant, revealing a significant decrease in the ease of learning ratings (Estimate = $-0.8611$, SE = 0.3469, $z = -2.482$, $p = 0.0131$, CI = $[-1.54, -0.183]$). The "No Experience" category was defined by including participants who reported no experience with any of the listed AI automotive technologies (see Experience for the questions). Specifically, this category comprises 257 participants, while 221 participants reported having experience with these technologies. This suggests that females with no experience find it significantly harder to learn to use automotive AI compared to their male counterparts with no experience. This model included only 471 observations, excluding data points from the "Other" category due to insufficient sample size, with a log-likelihood of $-597.38$ and an AIC of 1208.77. The model converged after six iterations, with a maximum gradient of $1.61 \times 10^{-13}$ and a condition number of 57.

#### Question 2: Using automotive AI would improve my performance at accomplishing driving-related tasks

The model had a log-likelihood of $-687.65$ and an AIC of 1387.30. The coefficient for the `Female` variable was estimated at $-0.2253$ with a standard error of 0.1672, resulting in a $z$-value of $-1.348$ and a $p$-value of 0.178, indicating that the effect of gender on performance improvement is not statistically significant. The coefficient for the `Other` variable was estimated at $-0.1820$ with a standard error of 0.7251, resulting in a $z$-value of $-0.251$ and a $p$-value of 0.802, also indicating no significant effect. The 95% confidence intervals for these estimates ranged from $-0.554$ to 0.102 for `Female` and from $-1.62$ to 1.28 for `Other`.

#### Question 3: Using automotive AI is something I would do in the future or will continue to do so.

The model had a log-likelihood of $-699.62$ and an AIC of 1411.24. The coefficient for the `Female` variable was estimated at $-0.5015$ with a standard error of 0.1678, resulting in a $z$-value of $-2.988$ and a $p$-value of 0.0028, indicating a statistically significant negative effect of being female on the intention to use automotive AI. The coefficient for the `Other` variable was estimated at $-1.3177$ with a standard error of 0.7724, resulting in a $z$-value of $-1.706$ and a $p$-value of 0.0880, indicating a marginally significant negative effect for the combined "Other" category. The 95% confidence intervals for these estimates ranged from $-0.832$ to $-0.174$ for `Female` and from $-2.82$ to 0.290 for `Other`.

### Question 4: Automotive AI technologies are competent in their area of expertise

The model had a log-likelihood of −621.03 and an AIC of 1254.05. The coefficient for the `Female` variable was estimated at −0.2867 with a standard error of 0.1717, resulting in a $z$-value of −1.670 and a $p$-value of 0.0949, indicating a marginally significant negative effect of being female on the perception of AI competence. The coefficient for the `Other` variable was estimated at −0.6836 with a standard error of 0.7779, resulting in a $z$-value of −0.879 and a $p$-value of 0.3795, indicating no significant effect for the combined "Other" category. The 95% confidence intervals for these estimates ranged from −0.624 to 0.0492 for `Female` and from −2.20 to 0.927 for `Other`.

### Question 5: Automotive AI technologies care about our well-being

The model had a log-likelihood of −716.91 and an AIC of 1445.81. The coefficient for the `Female` variable was estimated at −0.2455 with a standard error of 0.1654, resulting in a $z$-value of −1.484 and a $p$-value of 0.138, indicating that the effect of gender on the perception of information abuse is not statistically significant. The coefficient for the `Other` variable was estimated at −1.0647 with a standard error of 0.7535, resulting in a $z$-value of −1.413 and a $p$-value of 0.158, also indicating no significant effect for the combined "Other" category. The 95% confidence intervals for these estimates ranged from −0.570 to 0.0783 for `Female` and from −2.58 to 0.457 for `Other`.

### Question 6: Automotive AI technologies do not abuse the information and advantage they have over their users

The model had a log-likelihood of −666.04 and an AIC of 1344.08. The coefficient for the `Female` variable was estimated at −0.1793 with a standard error of 0.1686, resulting in a $z$-value of −1.064 and a $p$-value of 0.288, indicating that the effect of gender on trust in AI is not statistically significant. The coefficient for the `Other` variable was estimated at −0.6749 with a standard error of 0.7119, resulting in a $z$-value of −0.948 and a $p$-value of 0.343, also indicating no significant effect for the combined "Other" category. The 95% confidence intervals for these estimates ranged from −0.510 to 0.151 for `Female` and from −2.10 to 0.745 for `Other`.

### Question 7: I trust that automotive AI can offer information and service that is in my best interest

The model had a log-likelihood of −646.52 and an AIC of 1305.03. The coefficient for the `Female` variable was estimated at −0.2345 with a standard error of 0.1712, resulting in a $z$-value of −1.369 and a $p$-value of 0.1709, indicating that the effect of gender on positive feelings toward AI is not statistically significant. The coefficient for the `Other` variable was estimated at −1.4584 with a standard error of 0.7831, resulting in a $z$-value of −1.862 and a $p$-value of 0.0625, indicating a marginally significant negative effect for the combined "Other" category. The 95% confidence intervals for these estimates ranged from −0.571 to 0.101 for `Female` and from −2.98 to 0.182 for `Other`.

### Question 8: I feel positive toward automotive AI technologies

The model had a log-likelihood of −683.79 and an AIC of 1379.58. The coefficient for the `Female` variable was estimated at −0.5831 with a standard error of 0.1695, resulting in a $z$-value of −3.439 and a $p$-value of 0.000583, indicating a statistically significant negative effect of being female on the perception of AI trustworthiness. The coefficient for the `Other` variable was estimated at −1.6211 with a standard error of 0.7767,

resulting in a $z$-value of $-2.087$ and a $p$-value of 0.036871, indicating a statistically significant negative effect for the combined "Other" category. The 95% confidence intervals for these estimates ranged from $-0.917$ to $-0.252$ for `Female` and from $-3.13$ to $-0.00372$ for `Other`.

## Age

We conducted ordinal regression analyses to examine the relationship between age groups and responses to various questions about AI. The analysis included three age groups: 68 participants aged 18-30, 240 participants aged 30-45, 166 participants aged 45-65, and 3 participants who preferred not to disclose their age. The results for each main question are summarized below.

### AI Accountability

The analysis for the "AI Accountability" question encountered issues with convergence. Specifically, the model fitting process produced NaN values for standard errors, statistics, and confidence intervals. This issue indicates instability in parameter estimation, likely due to a highly significant deviation from a uniform distribution of responses. A chi-square goodness-of-fit test confirmed that the response distribution for this question significantly deviated from a uniform distribution ($X^2 = 438.24$, df = 4, $p < 2.2 \times 10^{-16}$). This non-uniform distribution contributed to the model's inability to accurately estimate parameters for the age groups. Consequently, no reliable estimates or confidence intervals could be reported for the "AI Accountability" question.

### AI Blameworthiness

For the "AI Blameworthiness" question, the age group 30-45 years old had a significant negative association with the response (estimate = $-0.510$, SE = 0.237, $z = -2.15$, $p = 0.031$, 95% $CI = [-0.974, -0.0456]$), indicating that this age group was less likely to attribute blameworthiness to AI compared to the reference group (18-30 years old). Similarly, the age group 45-65 years old also showed a significant negative association (estimate = $-0.665$, SE = 0.277, $z = -2.40$, $p = 0.016$, 95% $CI = [-1.21, -0.123]$).

### Confidence in Driving

For the "Confidence in Driving" question, neither the age group 30-45 years old (estimate = 0.587, SE = 0.429, $z = 1.37$, $p = 0.171$, 95% $CI = [-0.254, 1.43]$) nor the age group 45-65 years old (estimate = 0.00446, SE = 0.449, $z = 0.00993$, $p = 0.992$, 95% $CI = [-0.875, 0.884]$) showed significant associations with the response.

### Confidence in Learning

For the "Confidence in Learning" question, the age group 30-45 years old exhibited a significant positive association with the response (estimate = 1.34, SE = 0.474, $z = 2.83$, $p = 0.00469$, 95% $CI = [0.411, 2.27]$), indicating higher confidence in AI learning capabilities. The age group 45-65 years old did not show a significant association (estimate = 0.184, SE = 0.493, $z = 0.373$, $p = 0.709$, 95% $CI = [-0.782, 1.15]$).

The analysis highlights distinct age-related differences in perceptions of AI accountability, blameworthiness, and confidence in AI applications. For "AI Blameworthiness," both the 30-45 (estimate = $-0.510$, $p = 0.031$) and 45-65 (estimate = $-0.665$, $p = 0.016$) age groups were significantly less likely to attribute blame to AI compared to the 18-30 age group. In contrast, age did not significantly impact

"Confidence in Driving." However, for "Confidence in Learning," the 30-45 age group exhibited a significant positive association (estimate = 1.34, $p = 0.00469$), indicating higher confidence in AI learning capabilities. These findings underscore that age significantly influences perceptions of AI blameworthiness and learning confidence, while its impact on driving confidence is not significant.

### Technology Acceptance Questions

These results do not include Question 3 "Using automotive AI is something I would do in the future or will continue to do so.", as this is discussed above under Intention to Use.

#### Question 1: Learning to use automotive AI would be easy for me

The model included 478 observations, with a log-likelihood of $-628.39$ and an AIC of 1270.77. The model converged after six iterations, with a maximum gradient of $3.91 \times 10^{-13}$ and a condition number of 192. For the age group 30-45 years old, the estimate was 0.5453 (SE = 0.2569, $z = 2.122$, $p = 0.0338$, 95% $CI = [0.0413, 1.05]$), indicating a significant effect. For the age group 45-65 years old, the estimate was 0.2459 (SE = 0.2681, $z = 0.917$, $p = 0.3590$, 95% $CI = [-0.280, 0.772]$). For those who preferred not to say their age, the estimate was $-1.3716$ (SE = 0.9424, $z = -1.455$, $p = 0.1456$, 95% $CI = [-3.31, 0.519]$).

#### Question 2: Using automotive AI would improve my performance at accomplishing driving-related tasks

The model included 478 observations, with a log-likelihood of $-686.95$ and an AIC of 1387.89. The model converged after five iterations, with a maximum gradient of $2.25 \times 10^{-8}$ and a condition number of 210. For the age group 30-45 years old, the estimate was $-0.1411$ (SE = 0.2541, $z = -0.555$, $p = 0.5788$, 95% $CI = [-0.641, 0.357]$). For the age group 45-65 years old, the estimate was $-0.1599$ (SE = 0.2688, $z = -0.595$, $p = 0.5520$, 95% $CI = [-0.688, 0.367]$). For those who preferred not to say their age, the estimate was $-1.6808$ (SE = 0.9138, $z = -1.839$, $p = 0.0659$, 95% $CI = [-3.53, 0.180]$).

#### Question 3: Using automotive AI is something I would do in the future or will continue to do so.

The model included 478 observations, with a log-likelihood of $-699.54$ and an AIC of 1413.09. The model converged after five iterations, with a maximum gradient of $1.24 \times 10^{-9}$ and a condition number of 230. For the age group 30-45 years old, the estimate was 0.3453 (SE = 0.2492, $z = 1.386$, $p = 0.1659$). For the age group 45-65 years old, the estimate was $-0.1084$ (SE = 0.2584, $z = -0.420$, $p = 0.6748$). For those who preferred not to say their age, the estimate was $-1.7513$ (SE = 0.8848, $z = -1.979$, $p = 0.0478$). The results indicate that age did not significantly impact the intention to use automotive AI for most age groups, except for those who preferred not to disclose their age (three participants), who showed a significant negative association.

#### Question 4: Automotive AI technologies are competent in their area of expertise

The model included 478 observations, with a log-likelihood of $-620.11$ and an AIC of 1254.21. The model converged after six iterations, with a maximum gradient of $3.42 \times 10^{-13}$ and a condition number of 230. For the age group 30-45 years old, the estimate was 0.2917 (SE = 0.2598, $z = 1.123$, $p = 0.2614$, 95% $CI = [-0.219, 0.801]$).

For the age group 45-65 years old, the estimate was 0.2389 (SE = 0.2742, $z = 0.871$, $p = 0.3847$, 95% $CI = [-0.297, 0.774]$). For those who preferred not to say their age, the estimate was $-1.7949$ (SE = 1.0631, $z = -1.688$, $p = 0.0913$, 95% $CI = [-4.06, 0.275]$).

#### Question 5: Automotive AI technologies care about our well-being

The model included 478 observations, with a log-likelihood of $-716.66$ and an AIC of 1447.31. The model converged after five iterations, with a maximum gradient of $7.36 \times 10^{-9}$ and a condition number of 290. For the age group 30-45 years old, the estimate was $-0.2750$ (SE = 0.2503, $z = -1.099$, $p = 0.2720$, 95% $CI = [-0.768, 0.215]$). For the age group 45-65 years old, the estimate was $-0.2872$ (SE = 0.2629, $z = -1.093$, $p = 0.2746$, 95% $CI = [-0.804, 0.227]$). For those who preferred not to say their age, the estimate was $-1.8193$ (SE = 0.9600, $z = -1.895$, $p = 0.0581$, 95% $CI = [-3.92, 0.0660]$).

#### Question 6: Automotive AI technologies do not abuse the information and advantage they have over their users

The model included 478 observations, with a log-likelihood of $-663.44$ and an AIC of 1340.87. The model converged after five iterations, with a maximum gradient of $1.72 \times 10^{-7}$ and a condition number of 190. For the age group 30-45 years old, the estimate was 0.4084 (SE = 0.2619, $z = 1.559$, $p = 0.1189$, 95% $CI = [-0.105, 0.923]$). For the age group 45-65 years old, the estimate was 0.6435 (SE = 0.2741, $z = 2.348$, $p = 0.0189$, 95% $CI = [0.107, 1.18]$), indicating a significant effect. For those who preferred not to say their age, the estimate was $-0.6537$ (SE = 0.9484, $z = -0.689$, $p = 0.4906$, 95% $CI = [-2.55, 1.28]$).

#### Question 7: I trust that automotive AI can offer information and service that is in my best interest

The model included 478 observations, with a log-likelihood of $-646.03$ and an AIC of 1306.05. The model converged after six iterations, with a maximum gradient of $1.08 \times 10^{-13}$ and a condition number of 260. For the age group 30-45 years old, the estimate was 0.1024 (SE = 0.2622, $z = 0.390$, $p = 0.6962$, 95% $CI = [-0.414, 0.615]$). For the age group 45-65 years old, the estimate was 0.0647 (SE = 0.2725, $z = 0.237$, $p = 0.8123$, 95% $CI = [-0.471, 0.598]$). For those who preferred not to say their age, the estimate was $-2.2544$ (SE = 1.0182, $z = -2.214$, $p = 0.0268$, 95% $CI = [-4.44, -0.257]$), indicating a significant effect.

#### Question 8: I feel positive toward automotive AI technologies

The model included 478 observations, with a log-likelihood of $-687.67$ and an AIC of 1389.34. The model converged after five iterations, with a maximum gradient of $1.19 \times 10^{-7}$ and a condition number of 300. For the age group 30-45 years old, the estimate was 0.0266 (SE = 0.2546, $z = 0.104$, $p = 0.9170$, 95% $CI = [-0.474, 0.525]$). For the age group 45-65 years old, the estimate was $-0.1137$ (SE = 0.2666, $z = -0.426$, $p = 0.6699$, 95% $CI = [-0.638, 0.408]$). For those who preferred not to say their age, the estimate was $-2.4890$ (SE = 0.9929, $z = -2.507$, $p = 0.0122$, 95% $CI = [-4.63, -0.539]$), indicating a significant effect.

In summation, significant age-related effects were observed in several questions. Participants aged 30-45 found it significantly easier to learn automotive AI ($p = 0.0338$). The 45-65 age group showed significantly higher trust that automotive AI would not abuse its advantages ($p = 0.0189$). These findings suggest that younger and middle-aged

adults are more accepting of automotive AI. Additionally, participants who preferred not to say their age showed significantly lower trust that automotive AI can offer information and service in their best interest ($p = 0.0268$) and felt significantly less positive toward automotive AI technologies ($p = 0.0122$).

## Experience

We conducted ordinal regression analyses to examine the relationship between the experience of respondents and their responses to different questions regarding AI. The "No Experience" category was defined by including participants who reported no experience with any of the listed AI automotive technologies (see Experience for the exact questions). Specifically, this category comprises 257 participants, while 221 participants reported having experience with these technologies. The analysis compared individuals with no experience versus those with experience with automotive AI. The results are summarized below for each main question.

### AI Accountability

For the "AI Accountability" question, respondents with no experience with AI showed a significant positive association with the response (estimate = 0.918, SE = 0.338, $z = 2.71$, $p = 0.007$, 95% CI = $[0.255, 1.58]$). This indicates that individuals without AI experience were more likely to have a higher level of accountability assigned to AI compared to those with AI experience.

### AI Blameworthiness

Similarly, for the "AI Blameworthiness" question, respondents with no experience with AI also showed a significant positive association with the response (estimate = 0.865, SE = 0.313, $z = 2.77$, $p = 0.006$, 95% CI = $[0.252, 1.48]$). This suggests that those without AI experience were more likely to attribute blameworthiness to AI compared to those with AI experience.

### Confidence in Driving

In contrast, for the "Confidence in Driving" question, respondents with no experience with AI exhibited a significant negative association with the response (estimate = $-0.740$, SE = 0.287, $z = -2.58$, $p = 0.010$, 95% CI = $[-1.30, -0.177]$). This indicates that individuals without AI experience had lower confidence in AI driving capabilities compared to those with AI experience.

### Confidence in Learning

For the "Confidence in Learning" question, respondents with no experience with AI again showed a significant negative association with the response (estimate = $-1.00$, SE = 0.320, $z = -3.13$, $p = 0.002$, 95% CI = $[-1.63, -0.376]$). This suggests that those without AI automotive experience had lower confidence in AI learning capabilities compared to those with experience.

The analysis revealed distinct patterns in attitudes toward AI based on experience. Respondents with no experience with AI assigned significantly higher levels of accountability and blameworthiness to AI, with estimates of 0.918 (SE = 0.338, $z = 2.71$, $p = 0.007$) and 0.865 (SE = 0.313, $z = 2.77$, $p = 0.006$) respectively, indicating a greater tendency to hold AI accountable and blameworthy. Conversely, these individuals displayed significantly lower confidence in AI's driving and learning

capabilities, reflected in negative estimates of $-0.740$ (SE = 0.287, $z = -2.58$, $p = 0.010$) and $-1.00$ (SE = 0.320, $z = -3.13$, $p = 0.002$), suggesting that lack of AI experience correlates with skepticism regarding AI's effectiveness in these domains. These findings underscore the impact of AI experience on perceptions of accountability, blameworthiness, and confidence in AI capabilities.

## Technology Acceptance Questions

These results do not include Question 3 "Using automotive AI is something I would do in the future or will continue to do so.", as this is discussed above under Intention to Use.

### Question 1: Learning to use automotive AI would be easy for me

The model included 478 observations, with a log-likelihood of $-622.10$ and an AIC of 1254.21. The estimate for participants with no experience was $-0.7957$ (SE = 0.1736, $z = -4.583$, $p < 0.001$, 95% CI = $[-1.14, -0.457]$), indicating a significant negative effect.

### Question 2: Using automotive AI would improve my performance at accomplishing driving-related tasks

The model included 478 observations, with a log-likelihood of $-681.40$ and an AIC of 1372.79. The estimate for participants with no experience was $-0.6345$ (SE = 0.1686, $z = -3.762$, $p < 0.001$, 95% CI = $[-0.967, -0.305]$), indicating a significant negative effect.

### Question 3: Using automotive AI is something I would do in the future or will continue to do so.

The model included 478 observations, with a log-likelihood of $-691.40$ and an AIC of 1392.79. The estimate for participants with no experience was $-0.8802$ (SE = 0.1709, $z = -5.151$, $p < 0.001$, 95% CI = $[-1.22, -0.547]$), indicating a significant negative effect for participants with no experience using automotive AI.

### Question 4: Automotive AI technologies are competent in their area of expertise

The model included 478 observations, with a log-likelihood of $-617.41$ and an AIC of 1244.81. The estimate for participants with no experience was $-0.557$ (SE = 0.173, $z = -3.22$, $p = 0.001$, 95% CI = $[-0.898, -0.219]$), indicating a significant negative effect.

### Question 5: Automotive AI technologies care about our well-being

The model included 478 observations, with a log-likelihood of $-713.86$ and an AIC of 1437.72. The estimate for participants with no experience was $-0.5179$ (SE = 0.1659, $z = -3.121$, $p = 0.002$, 95% CI = $[-0.844, -0.194]$), indicating a significant negative effect.

#### Question 6: Automotive AI technologies do not abuse the information and advantage they have over their users

The model included 478 observations, with a log-likelihood of −665.56 and an AIC of 1341.12. The estimate for participants with no experience was −0.279 (SE = 0.168, $z = -1.66$, $p = 0.097$, 95% CI = $[-0.609, 0.0499]$), indicating a non-significant effect.

#### Question 7: I trust that automotive AI can offer information and service that is in my best interest

The model included 478 observations, with a log-likelihood of −647.23 and an AIC of 1304.45. The estimate for participants with no experience was −0.3007 (SE = 0.1707, $z = -1.762$, $p = 0.078$, 95% CI = $[-0.636, 0.0332]$), indicating a non-significant effect.

#### Question 8: I feel positive toward automotive AI technologies

The model included 478 observations, with a log-likelihood of −685.25 and an AIC of 1380.50. The estimate for participants with no experience was −0.5721 (SE = 0.1688, $z = -3.39$, $p < 0.001$, 95% CI = $[-0.904, -0.243]$), indicating a significant negative effect.

## Discussion of Exploratory Analysis

The exploratory analysis highlighted several significant demographic effects on attitudes towards automotive AI, focusing on gender, age, and experience.

#### Gender

Gender was a notable factor influencing attitudes towards automotive AI. Female participants generally reported lower levels of AI accountability amd AI blameworthiness, suggesting female participants are more lenient toward AI than their male counterparts. Additionally, female participants generally reported lower confidence in driving with AI and confidence in learning to drive with AI compared to their male counterparts. Specifically, being female had a significant negative effect on the intention to use automotive AI, with a coefficient of −0.5015 (SE = 0.1678, $z = -2.988$, $p = 0.0028$). What is more, an interaction effect between being female and having no AI experience was significant for the ease of learning to use automotive AI (Estimate = −0.8611, SE = 0.3469, $z = -2.482$, $p = 0.0131$), indicating that females with no AI experience found it significantly more challenging compared to their male counterparts with no experience.

#### Age

Age significantly influenced perceptions of AI in various ways. Older participants were less likely to attribute blame or accountability to automotive AI. Specifically, the 30-45 and 45-65 age groups showed a lower likelihood of attributing blame to AI (estimates of −0.510, $p = 0.031$ and −0.665, $p = 0.016$, respectively) compared to the 18-30 age group. Conversely, the 30-45 age group reported higher confidence in AI learning capabilities (estimate = 1.34, $p = 0.00469$). However, age did not significantly affect confidence in driving.

#### Experience

Experience with AI was a strong determinant of attitudes towards automotive AI. Participants with no experience assigned significantly higher levels of accountability and

blameworthiness to AI, with estimates of 0.918 (SE = 0.338, $z = 2.71$, $p = 0.007$) and 0.865 (SE = 0.313, $z = 2.77$, $p = 0.006$), respectively. They also exhibited lower confidence in driving and learning to drive with AI, reflected in estimates of $-0.740$ (SE = 0.287, $z = -2.58$, $p = 0.010$) and $-1.00$ (SE = 0.320, $z = -3.13$, $p = 0.002$). These findings highlight that a lack of AI experience correlates with more negative perceptions, an increased likelihood to blame, and lower acceptance of AI technologies across various aspects, including ease of learning, perceived performance improvement, and overall positivity towards AI.

Finally, the analysis revealed no significant interaction effects between any demographic variables and the group label ('trustworthy AI'). This indicates that the relationship between the demographic factors and the respondents' perceptions was consistent across both the 'trustworthy AI' and 'reliable AI' groups.

**Conclusion**

The exploratory analysis highlights significant demographic effects on evaluations of automotive AI, providing further insight into the philosophical debate regarding AI trust. Specifically, the finding that female participants and participants with no experience report lower levels of confidence stands in stark contrast with the absence of interaction effects between group assignment and gender or experience. This suggests that the label "trustworthy AI" does improve the negative attitudes held by these participants. Moreover, since these participants also report lower ratings for intention to use, this further emphasizes the viewpoint that trustworthy AI is an inappropriate target for alleviating algorithm aversion. Consequently, the exploratory analysis indicates that demographic factors, particularly gender and experience, significantly influence perceptions of AI's trustworthiness and reliability. The negative association between being female and various trust metrics suggests that AI design and labeling strategies should be sensitive to these demographic differences. This finding supports the ethical argument that AI systems should be designed and communicated in ways that recognize and address the varied trust dynamics among users.

For developers and policymakers, the exploratory analysis provides crucial insights into how demographic factors shape user attitudes toward AI. The significant negative impact of being female on trust-related metrics implies that marketing and educational strategies should be tailored to address the specific concerns of female users. By focusing on reliability and transparency, developers can better manage user expectations and develop more inclusive acceptance of automotive AI technologies.

# References


1. Bryson J. AI & Global Governance: No One Should Trust AI. United Nations University, Centre for Policy Research, 2018. URL: https://unu.edu/cpr/blog-post/ai-global-governance-no-one-should-trust-ai.

2. Ryan M. In AI We Trust: Ethics, Artificial Intelligence, and Reliability. Science and Engineering Ethics, 26, 2749–2767, 2020. DOI: 10.1007/s11948-020-00228-y.

3. Dorsch J, Deroy O. Quasi-Metacognitive Machines: Why We Don't Need Morally Trustworthy AI and Communicating Reliability is Enough. Philosophy & Technology, 37, 62, 2024. DOI: 10.1007/s13347-024-00752-w.

4. Buechner J., Tavani H.T. Trust and multi-agent systems: applying the "diffuse, default model" of trust to experiments involving artificial agents. Ethics Inf Technol 13, 39–51 (2011). DOI: 10.1007/s10676-010-9249-z.

5. Coeckelbergh M. Can We Trust Robots? Ethics and Information Technology, 14(1), 53-60, 2012. DOI: 10.1007/s10676-011-9279-1.
6. Baier A. Trust and Antitrust. Ethics, 96(2), 231-260, 1986. DOI: 10.1086/292745.
7. Hardin R. Trust and Trustworthiness. Russell Sage Foundation, 2002. ISBN: 9780871543414.
8. Lahno B. On the Emotional Character of Trust. Ethical Theory and Moral Practice 4, 171–189 (2001). DOI: 10.1023/A:1011425102875.
9. Shank, D. B., DeSanti, A., & Maninger, T. When are artificial intelligence versus human agents faulted for wrongdoing? Moral attributions after individual and joint decisions. Information, Communication & Society, 22(5), 648-663 (2019). DOI: 10.1080/1369118X.2019.1568515.
10. Porsdam Mann, S., Earp, B. D., Nyholm, S., Danaher, J., Møller, N., Bowman-Smart, H., Hatherley, J., Koplin, J., Plozza, M., Rodger, D., Treit, P. V., Renard, G., McMillan, J., & Savulescu, J. Generative AI entails a credit–blame asymmetry. Nature Machine Intelligence, 5(5), 472-475 (2023). DOI: 10.1038/s42256-023-00653-1.
11. Longin L, Bahrami B, Deroy O. Intelligence brings responsibility - Even smart AI assistants are held responsible. iScience, 26(8), 107494, 2023. DOI: 10.1016/j.isci.2023.107494.
12. Davis F.D. Perceived Usefulness, Perceived Ease of Use, and User Acceptance of Information Technology. MIS Quarterly, 13(3), 319-340, 1989. DOI: 10.2307/249008.
13. Davis, F.D., Granić, A Technology Acceptance Model. 30 Years of TAM. Springer, 2024. DOI: 10.1007/978-3-030-45274-2
14. Choung, H., David, P., & Ross, A. Trust in AI and Its Role in the Acceptance of AI Technologies. International Journal of Human–Computer Interaction, 39(9), 1727–1739, 2022. DOI: 10.1080/10447318.2022.2050543.
15. Waytz A, Heafner J, Epley N. The Mind in the Machine: Anthropomorphism Increases Trust in an Autonomous Vehicle. Journal of Experimental Social Psychology, 52, 113-117, 2014. DOI: 10.1016/j.jesp.2014.01.005.
16. Ruijten, P. A. M., Terken, J. M. B., & Chandramouli, S. N. Enhancing Trust in Autonomous Vehicles through Intelligent User Interfaces That Mimic Human Behavior. Multimodal Technologies and Interaction, 2(4), 62. DOI: 10.3390/mti2040062.
17. Cheng, X., Zhang, X., Cohen, J., & Mou, J. Human vs. AI: Understanding the impact of anthropomorphism on consumer response to chatbots from the perspective of trust and relationship norms. Information Processing & Management, Volume 59, Issue 3, 2022. DOI: 10.1016/j.ipm.2022.102940.
18. de Visser, E. J., Monfort, S. S., McKendrick, R., Smith, M. A. B., McKnight, P. E., Krueger, F., & Parasuraman, R. Almost human: Anthropomorphism increases trust resilience in cognitive agents. Journal of Experimental Psychology: Applied, 22(3), 331–349. DOI: 10.1037/xap0000092.

19. Dietvorst, B. J., Simmons, J. P., & Massey, C. Algorithm aversion: People erroneously avoid algorithms after seeing them err. Journal of Experimental Psychology: General, 144(1), 114-126. DOI: 10.1037/xge0000033.

20. Deroy, O. The Ethics of Terminology: Can we use human terms to describe AI? Topoi, 42(3), 881-889. DOI: 10.1007/s11245-023-09934-1

21. Inie, N., Druga, S., Zukerman, P., & Bender, E. M. From "AI" to Probabilistic Automation: How Does Anthropomorphization of Technical Systems Descriptions Influence Trust? In The 2024 ACM Conference on Fairness, Accountability, and Transparency (pp. 2322-2347). DOI: 10.1145/3630106.3659040.

22. Karpus, J., Krüger, A., Verba, J. T., Bahrami, B., & Deroy, O. Algorithm exploitation: Humans are keen to exploit benevolent AI. Iscience, 24(6). DOI: 10.1016/j.isci.2021.102679
